# A Physically Informed QUBO Model for π‑Stacking Interactions in Molecular Docking Problems

**DOI:** 10.1021/acsomega.6c02707

**Published:** 2026-06-26

**Authors:** Alessandro Beneventi, Anita Camillini, Francesco Micucci, Gabriella Bettonte, Matteo Barbieri, Gianluca Palermo, Andrea Rosario Beccari, Domenico Bonanni

**Affiliations:** † Scuola di Scienze, University of Bologna, Via Selmi 3, Bologna 40126, Italy; ‡ Quantum Computing Lab, 18216 CINECA Via Magnanelli, 2, Casalecchio di Reno 40033, Italy; § E4 Computer Engineering SpA, Viale Martiri della Libertà 66, Scandiano 42019, Italy; ∥ 18981 Politecnico di Milano, Via Giuseppe Ponzio 34/5, Milan 20133, Italy; ⊥ EXSCALATE, Dompé Farmaceutici S.p.A., Via Tommaso De Amicis 95, Naples 80131, Italy; # Department of Physical and Chemical Sciences, University of L’Aquila, Via Vetoio, Coppito, L’Aquila 67100, Italy; ¶ Data Science and Computation Facility, Istituto Italiano di Tecnologia, Via Morego 30, Genoa 16163, Italy

## Abstract

Molecular docking is a computational technique central to computer-aided drug discovery (CADD), aimed at predicting the preferred binding mode and binding affinity of a small-molecule ligand within the active site of a target macromolecule. Recent advances in quantum computing have led to the development of new molecular docking models. In particular, quantum annealing has motivated the formulation of physically informed docking frameworks that can be encoded within discrete optimization paradigms such as QUBO formulations.

## Graphical abstract

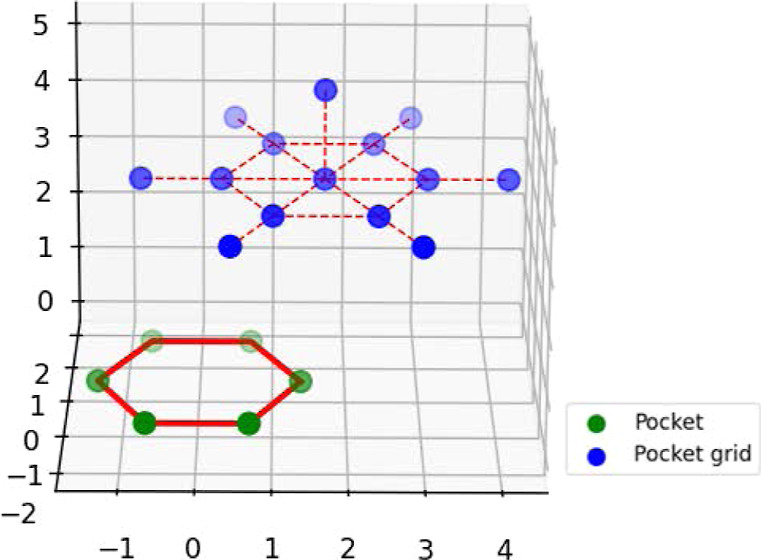

This work introduces a new Hamiltonian term that captures π-stacking interactions between aromatic systems, a class of noncovalent forces essential in biomolecular recognition yet often oversimplified in traditional docking approaches. High-level computational chemistry calculations were performed to obtain reference interaction energy profiles for benzene dimers, which were then used to guide the formulation of the proposed term.

The extended model was tested through simulated annealing on systems involving benzene and aromatic amino-acid residues (tyrosine, phenylalanine, and tryptophan). The results show that the new term consistently drives the optimization toward correct binding conformations, demonstrating the importance of explicitly incorporating π-stacking effects in discrete docking formulations and supporting future developments in quantum-enhanced molecular docking.

## Introduction

Molecular docking^1–3^ is a key computational technique in structural biology and drug discovery,^4^ used to predict the preferred three-dimensional arrangement of a ligand bound to its biological target and to estimate the strength of their interaction. Its ability to virtually screen large chemical libraries and to provide insight into molecular recognition processes makes docking a fundamental component of modern computational chemistry.

As docking applications continue to grow in scale and complexity, there is increasing interest in improving the accuracy of scoring models while exploring computational paradigms capable of efficiently handling the large number of possible molecular configurations. Among these, quantum computing^5^ has emerged as a particularly promising framework for energy-based optimization problems.^6–10^ In particular, quantum annealing has shown encouraging performance on complex combinatorial optimization tasks, including molecular docking,^11–13^ although its practical advantage over classical methods for large and complex molecular systems remains an open and actively investigated question, as current hardware is still maturing toward the scale and precision required for chemically relevant applications. Quantum annealing leverages intrinsically quantum mechanical phenomena—namely tunneling and superposition—to efficiently explore the energy landscape of an optimization problem in search of low-energy solutions. Among the leading hardware implementations, D-Wave systems currently represent one of the most mature and widely adopted quantum annealing platforms, providing access to thousands of qubits through their quantum processing units. Quantum annealers naturally require the problem to be formulated in the quadratic unconstrained binary optimization (QUBO)^14^ framework, in which binary variables and their pairwise quadratic interactions collectively define the energy landscape to be minimized.

Formally, given a symmetric matrix $Q\in R^{n\times n}$ , the objective is to find a binary vector *x* ∈ {0,1}^*n*^ minimizing the cost function

$$f_{Q}\left(x\right)=x^{Τ}Qx$$

Within this framework, molecular docking can be recast as a QUBO problem by encoding the geometric^11^ and physicochemical constraints governing ligand–receptor interactions into a discrete Hamiltonian. This formulation establishes a direct correspondence between molecular configurations and binary variables, rendering the problem amenable to quantum annealing and enabling systematic exploration of high-dimensional docking landscapes beyond the reach of purely classical heuristics.

The present work builds upon the geometric framework for molecular docking introduced by Triuzzi et al.,^11^ where the docking problem is reformulated as a combinatorial graph-matching task suitable for quantum annealers. In that formulation, only geometric compatibility between ligand and pocket is encoded. A subsequent line of research,^15^ developed on top of this geometric foundation, extended the model by incorporating physicochemical interaction terms into the Hamiltonian. Within this combined geometric-physicochemical setting, both the ligand and the binding pocket are represented as weighted graphs, and the docking problem is cast as the search for an optimal correspondence between their nodes.

To enable a physically grounded description of π-stacking,^16–21^ extensive quantum chemistry calculations were performed on HPC infrastructures to characterize the interaction energy across a range of relative orientations and spatial displacements between benzene rings. These calculations, conducted using ab initio^22^ techniques and constructed following the methodology introduced by Schramm et al.,^23^ revealed the dominant features of the underlying potential energy surface (PES) and provided the quantitative reference data needed to construct an effective model.

Incorporating this interaction into a QUBO-based Hamiltonian required addressing geometric limitations inherent to atom-based graph representations. While the original formulation controls the placement of individual atoms, π-stacking depends on the collective orientation of an entire aromatic ring. Analysis of the computed energy surfaces showed that one degree of freedom could be safely neglected, making it possible to design a simplified yet accurate term that captures the essential physics while remaining compatible with the discrete optimization framework.

The resulting Hamiltonian term was validated through simulated annealing^24^ experiments involving benzene interacting with aromatic amino-acid residues such as tyrosine, phenylalanine, and tryptophan. Simulated annealing is a stochastic optimization technique in which candidate configurations are iteratively updated and accepted according to a temperature-dependent criterion, allowing the system to escape local minima and progressively converge toward low-energy states. In this context, a “sweep” denotes a set of move attempts performed at a fixed temperature, while a “read” corresponds to a complete annealing run from high to low temperature. Across a variety of grid resolutions and pocket geometries, the inclusion of the π-stacking contribution consistently guided the optimization toward physically meaningful binding orientations. These results demonstrate that explicitly including π-stacking interaction terms in system’s Hamiltonian, can substantially improve the predictive capabilities of discrete docking formulations and support the development of more realistic, physically informed models for molecular recognition.

### Previous Framework: Graph-Based Quantum Docking

The present work builds upon an hybrid geometric-physicochemical framework for molecular docking introduced by Triuzzi et al.,^11^ where the docking problem is reformulated as a combinatorial graph-matching task suitable for being straightforwardly executed on quantum annealers. The central idea is to encode both the ligand and the binding pocket as weighted graphs and to search for an energetically optimal correspondence between their nodes.

### Pocket Representation

The binding pocket is discretized into a collection of representative points —referred to as pocket probes—which encode information related to pocket’s accessible volume, steric shape and pharmacophoric properties. These probes define the nodes of a spatial grid graph

$$G_{grid}=\left\{N_{grid},E_{grid},W_{grid}^{N},W_{grid}^{E}\right\}$$

*N*_grid_: grid points inside the pocket that the ligand may occupy. *E*_grid_: edges connecting the points previously introduced. *W*_grid_^E^: euclidean distances between neighboring probes. *W*_grid_^N^: precomputed physicochemical descriptors (electrostatic potential, Lennard–Jones fields, etc.) encoding how the protein influences each spatial location.

Thus, each grid node represents not only a geometric position but also a local physicochemical environment generated by the protein.

### Ligand Representation

The ligand is represented by the weighted molecular graph

$$G_{mol}=\left\{N_{mol},E_{mol},W_{mol}^{N},W_{mol}^{E}\right\}$$

where *N*_mol_: ligand atoms (possibly after preprocessing such as removal of terminal hydrogens). *E*_mol_: chemical bonds between atoms, together with the additional edges introduced to constrain and model the geometric structure of the ligand. *W*_mol_^E^: bond-length constraints enforcing ligand geometry. *W*_mol_^N^: atom-level attributes such as partial charges and van der Waals type.

This representation encodes the chemical properties and preserves the three-dimensional structure of the ligand.

### General Structure of the Hamiltonian

In this framework, docking is solved by minimizing a global QUBO Hamiltonian of the form

$$H=\lambda_{geom}H_{geom}+\lambda_{el}H_{el}+\lambda_{vdw}H_{vdw}$$

where: *H*_geom_ enforces geometric and structural compatibility between ligand and grid (e.g., injective mapping, encoding of bond lengths, adherence to cavity geometry). *H*_el_ encodes electrostatic interactions using precomputed potentials. *H*_vdw_ models van der Waals interactions via Lennard-Jones descriptors.

Each term is expressed entirely in quadratic form in the variables *x*_*ii*′_ ∈ {0, 1}, where *x*_*ii*′_ = 1 if ligand atom *i* ∈ *G*_mol_ is assigned to grid point *i*′ ∈ *G*_grid_.

The tunable coefficients λ_geom_, λ_el_, λ_vdw_ control the relative importance of geometric fidelity and physicochemical forces.

This framework provides the mathematical and computational foundation upon which the present work builds. In particular, the new interaction term introduced in this article integrates seamlessly into the same graph-based QUBO structure, enriching the original formulation with an additional class of noncovalent interactions of central relevance in biomolecular docking.

### Theoretical Nature of the π-Stacking Interaction

π-Stacking interactions are a class of noncovalent forces occurring between aromatic systems such as benzene rings, polycyclic aromatic hydrocarbons, and aromatic residues in biomolecules. Initially rationalized through simplified electrostatic models^25,26^ , the understanding of π-stacking has progressively evolved with the development of modern quantum chemical methods that clarify the subtle balance between attractive dispersion forces and short-range repulsion^23,27^ . They arise from the interaction of delocalized π electrons: when two aromatic systems approach one another, their π-electron clouds can interact favorably, stabilizing specific stacked arrangements. These interactions play a central role in molecular recognition, protein folding, nucleic acid stability, and supramolecular self-assembly.

### Phenomenological Description and Energy Components

From a geometric standpoint, π-stacking interactions are commonly classified into three main configurations, illustrated by the benzene dimer in Figure 1. The inter-ring distances reported below are indicative, as small variations arise depending on the computational methods used to optimize the geometries.

- T-shaped, shown in Figure 1a, where one aromatic ring is oriented perpendicular to the other, giving rise to a characteristic “T” geometry. The distance between the center of one ring and the plane of the other is 5.0 Å.
- Parallel-displaced (slip-stacked), shown in Figure 1b, where the two rings are parallel but laterally shifted with respect to each other. This geometry balances stabilizing interactions while reducing excessive π–π overlap, and is characterized by an interplanar distance of 3.5 Å and a displacement of 1.7 Å, corresponding to a local energy minimum.
- Sandwich (cofacial), shown in Figure 1c, corresponding to a face-to-face arrangement with minimal lateral displacement. Despite maximizing π-orbital overlap, this configuration is energetically unfavorable due to strong short-range repulsion and represents a saddle point, with an interplanar separation of 3.8 Å.

**Figure 1:**
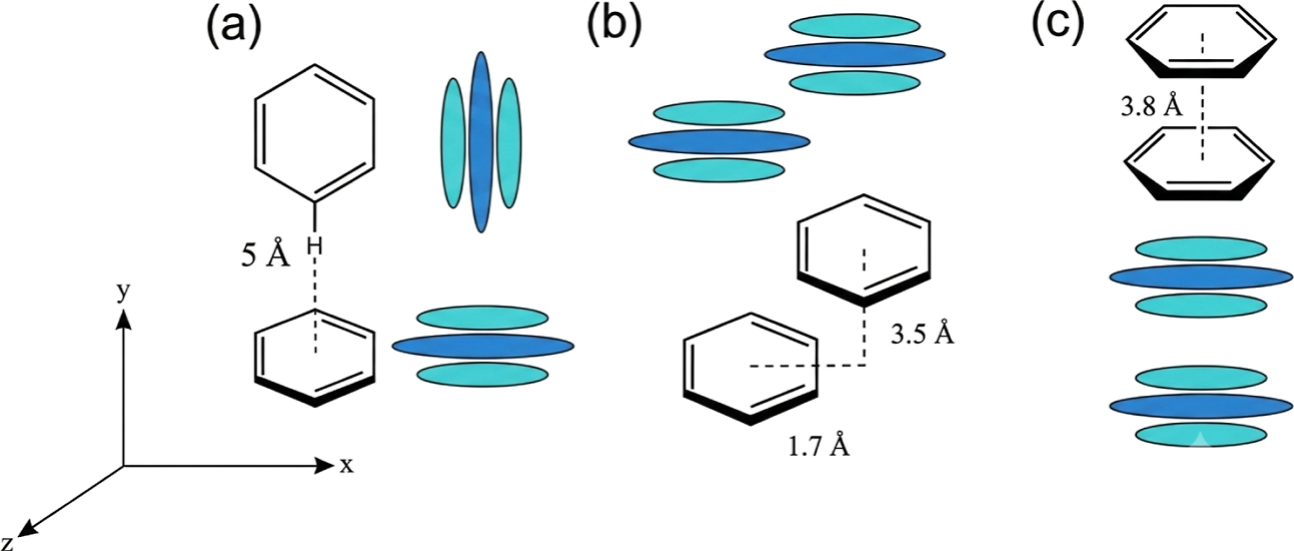
Three most common geometries of the benzene dimer: (a) T-shaped, (b) parallel-displaced, and (c) sandwich (cofacial). The color coding illustrates the electrostatic quadrupole: light blue (δ^–^) represents the π-electron clouds above and below the ring, while blue (δ^+^) indicates the partial positive charge at the molecular edge. Image adapted with permission from Schramm et al.^23^ Copyright 2025 American Chemical Society.

### Quantum-Chemistry Calculations

In this section, we present the results obtained from our quantum-chemistry calculations. Specifically, we computed the interaction energy between two benzene molecules as a function of their relative distance and orientation, in order to characterize the π-stacking interaction profile. The computational setup—comprising the input structure and the optimized atomic coordinates of the benzene monomers—was constructed starting from the reference geometries and interaction profiles reported by Schramm et al.^23^ Using these geometries as a baseline, we performed controlled rigid translations of one monomer to systematically investigate how the π-stacking interaction varies with intermolecular distance and relative orientation.

### Methods

To compute the interaction energy between two benzene rings, we employed the XSAPT + MBD method, which combines extended symmetry-adapted perturbation theory with a Many-Body Dispersion correction, enabling a physically transparent decomposition of the interaction energy into electrostatic, induction, dispersion, and exchange contributions. All calculations were carried out using the Q-Chem software package.^28^

All XSAPT calculations were performed using the LRC-ωPBE range-separated functional with the def2-TZVPD basis set, which includes diffuse and polarization functions suitable for accurately describing nonlocal interactions. The range–separation parameter ω = 0.34 bohr^–1^ was determined via the global density-dependent (GDD) tuning procedure. Dispersion interactions were treated using the many-body dispersion (MBD) correction, and all SAPT energy decompositions were carried out in the atomic orbital formalism up to second order.

Two SAPT calculations were performed for each system, differing in the treatment of electrostatic embedding. In the first, fragments are polarized by the point charges of the surrounding ones (embed charges), thus incorporating the effect of the electrostatic environment on the induction energy. In the second, embedding is disabled (embed none), providing a reference in which fragment polarization is treated in isolation.

Additionally, a δ_HF_ correction was computed via a dedicated calculation using the 6-311G basis set. This correction accounts for higher-order induction effects not captured by second-order perturbation theory, and is added to the induction energy to improve the overall accuracy of the interaction energy decomposition.

### Results and Discussion

Before presenting the results, we fix the reference frame used throughout this section: the *x*-axis denotes the direction along which lateral displacements between the two benzene molecules are applied; the *z*-axis corresponds to the vertical separation between the aromatic planes; and the *y*-axis is defined as the direction orthogonal to both *x* and *z*.

### Parallel-Displaced Configuration: Translations

We first analyzed rigid translations of one benzene ring in the parallel-displaced configuration. As shown in Figure 9a, the interaction energy depends strongly on both the lateral displacement *x* and the vertical separation *z* between the two molecular planes.

**Figure 2:**
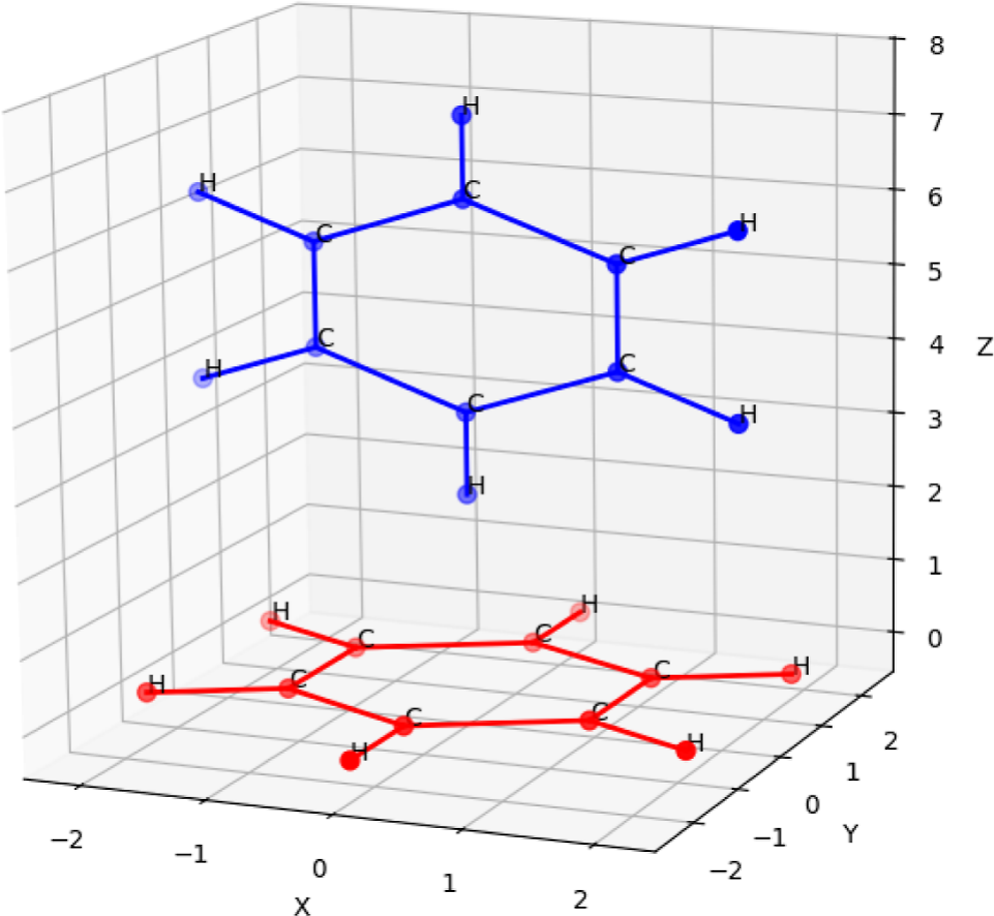
Spatial representation of the T-shaped configuration used in the calculations.

**Figure 3:**
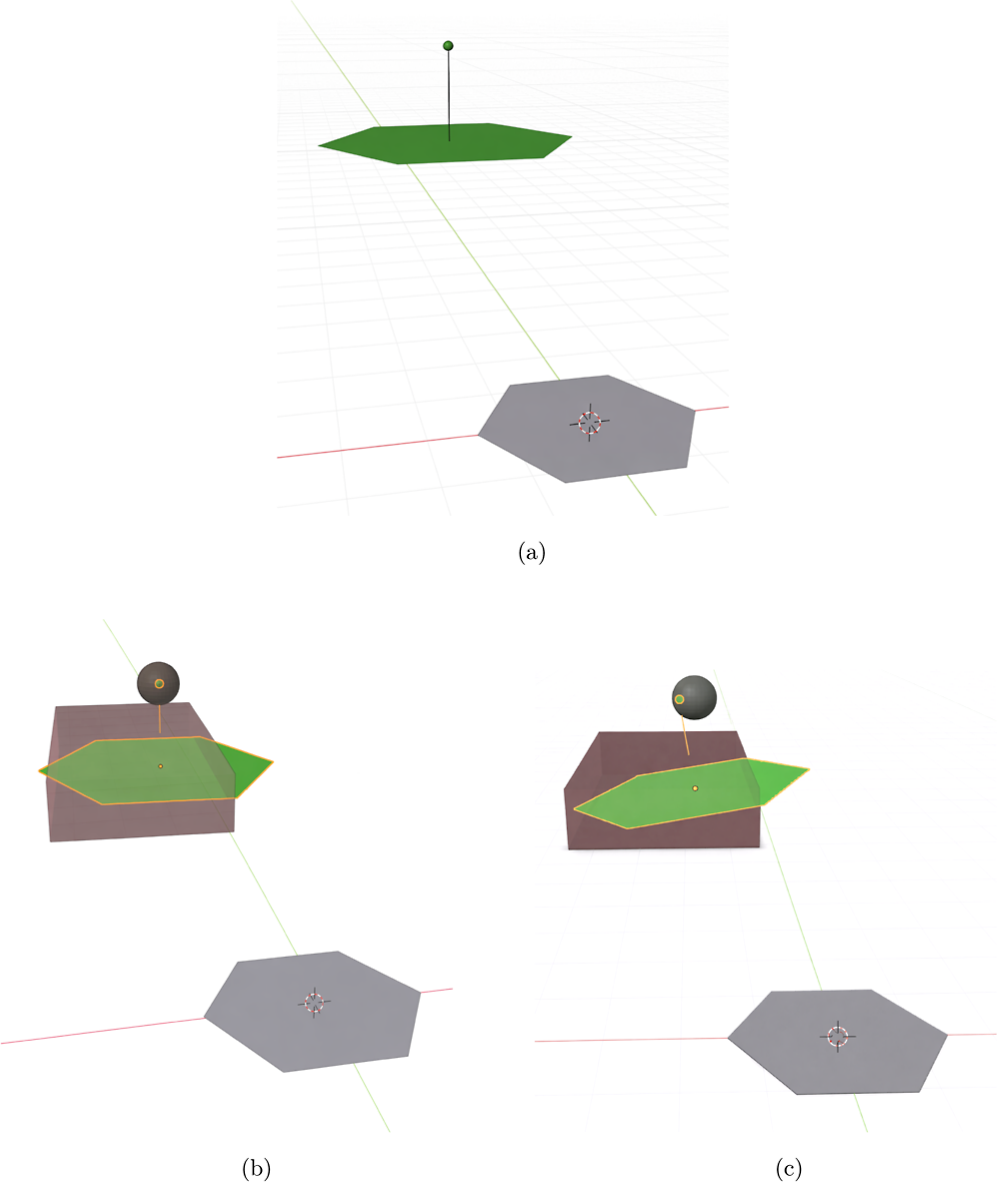
Figure illustrates the auxiliary construction performed in line with the modeling approach introduced. The gray hexagon represents an aromatic moiety within the protein, while the green fragment corresponds to the ligand’s aromatic moiety, with the two newly added nodes. The red cuboid indicates *G*_center_, and the black sphere represents the spherical surface corresponding to the admissible orientations.

**Figure 4:**
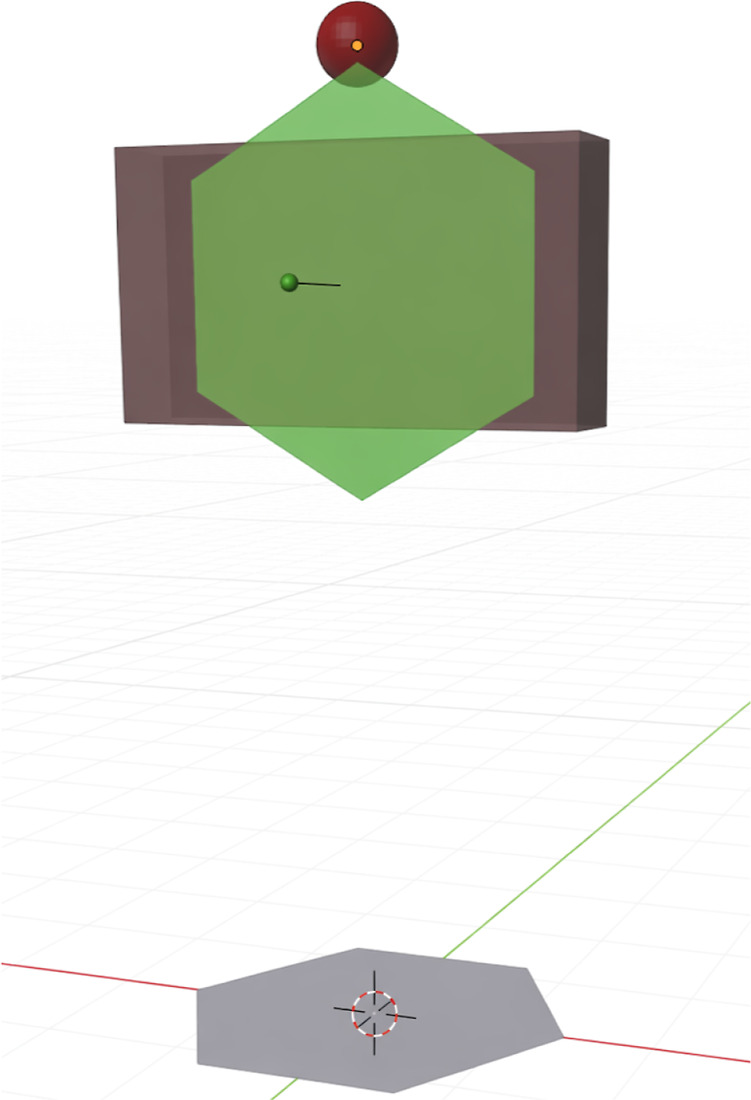
Representation of the T-shaped configuration with fixed vertical axis.

**Figure 5:**
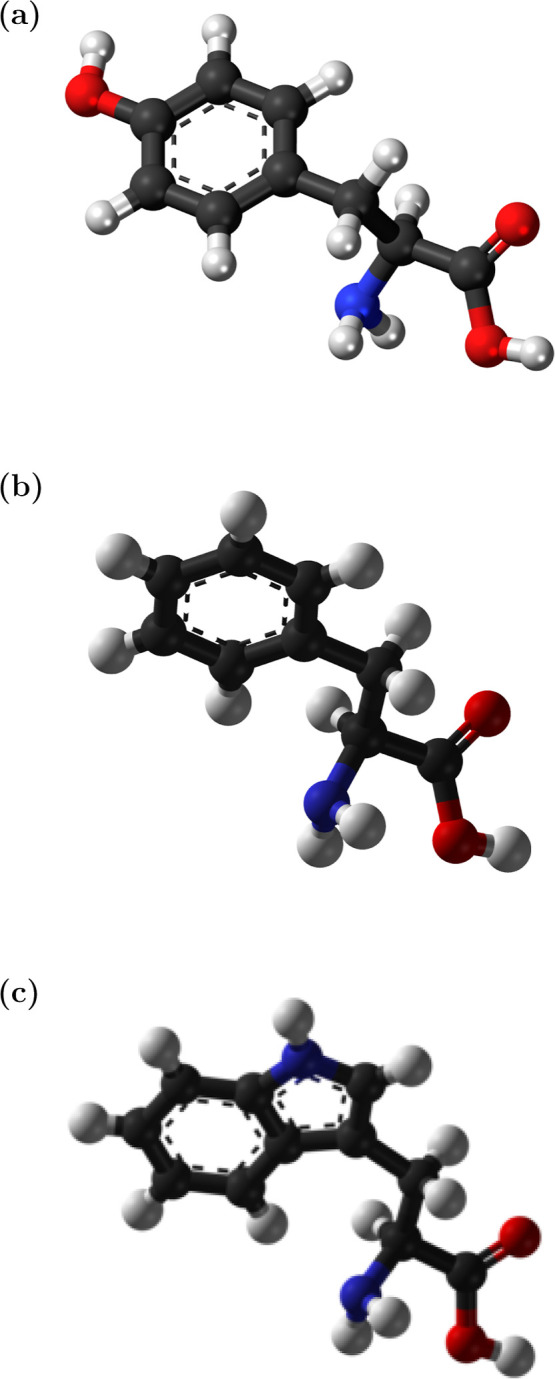
(a) Tyrosine (Tyr), C_9_H_11_NO_3_; (b) phenylalanine (Phe), C_9_H_11_NO_2_; (c) tryptophan (Trp), C_11_H_12_N_2_O. Images created using BIOVIA Discovery Studio Visualizer.

**Figure 6:**
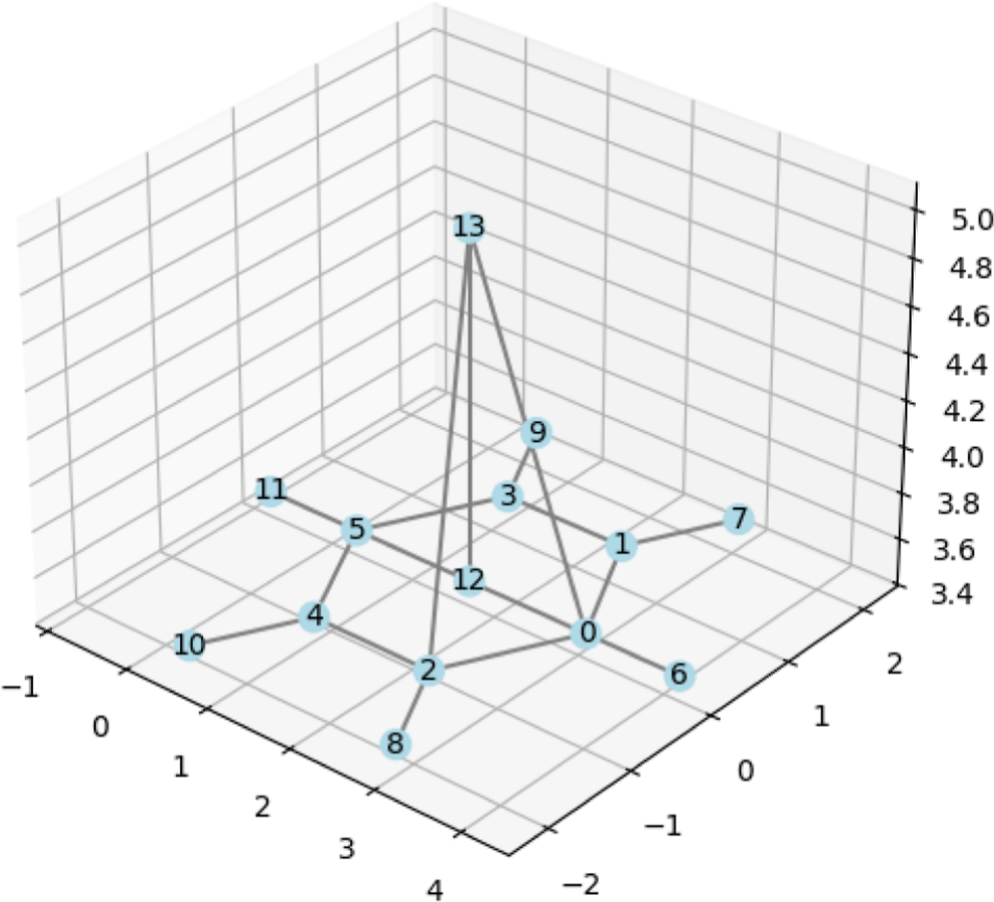
Benzene ligand with auxiliary nodes.

**Figure 7:**
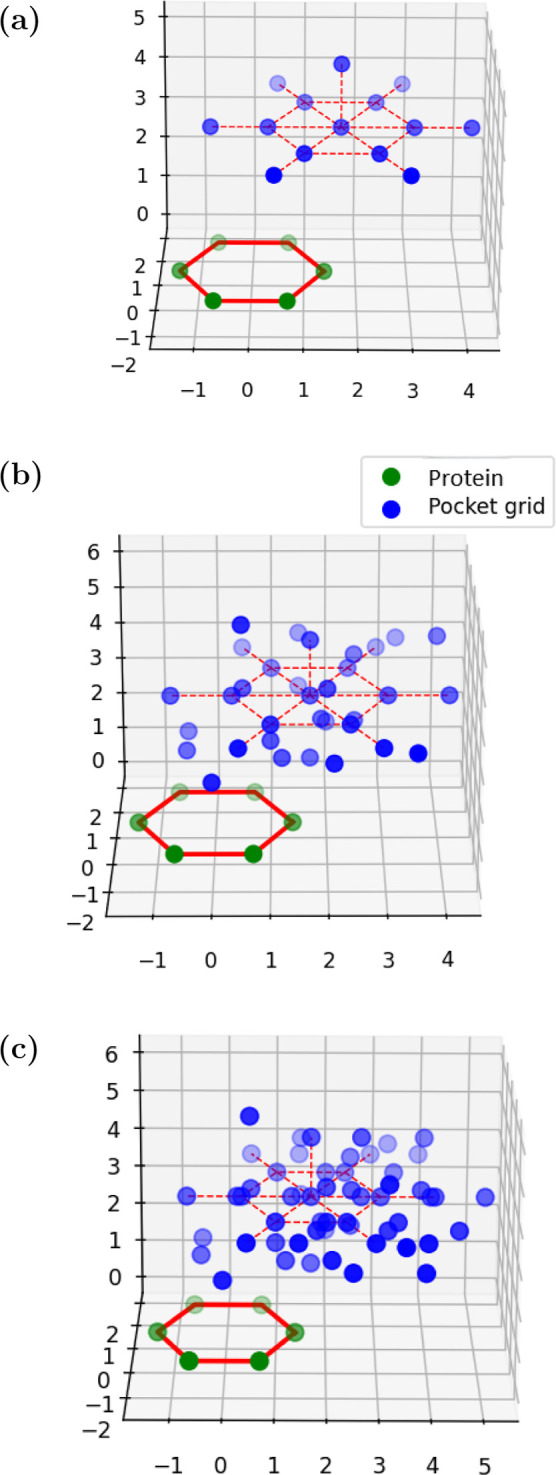
Pocket grid representations: (a) grid A, a minimal arrangement with 14 nodes to position the ligand in a single ideal pose; (b) grid B, grid A enriched with 26 additional nodes to allow more candidate ligand poses; (c) grid C, further expanded with an additional 14-node configuration to explore alternative binding geometries and increase spatial flexibility.

**Figure 8:**
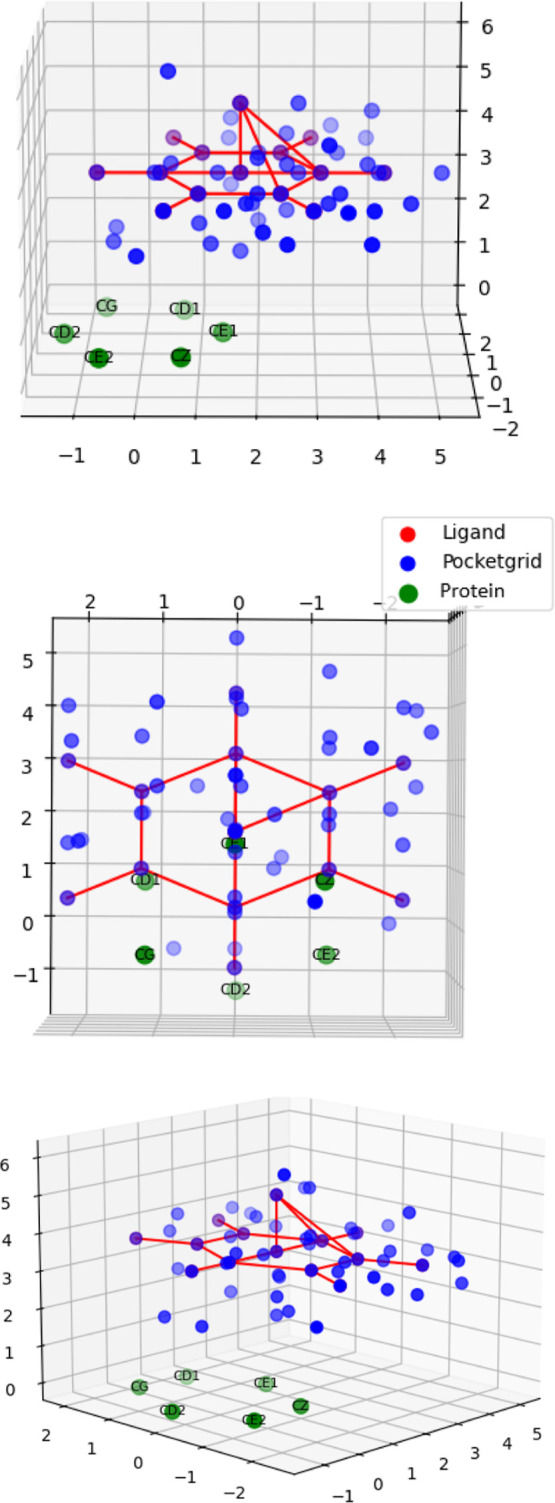
Ligand placement of a benzene molecule within pocket grid Grid C for the case λ_geom_ = 3, shown from three different viewing angles. Green nodes represent the benzene molecule used to define the pocket, light blue nodes indicate the pocket grid, and red nodes correspond to the ligand. The hydrogen atoms of the protein are included in the simulated annealing calculations but are omitted from the visualization for clarity and to avoid an overly crowded image.

**Figure 9:**
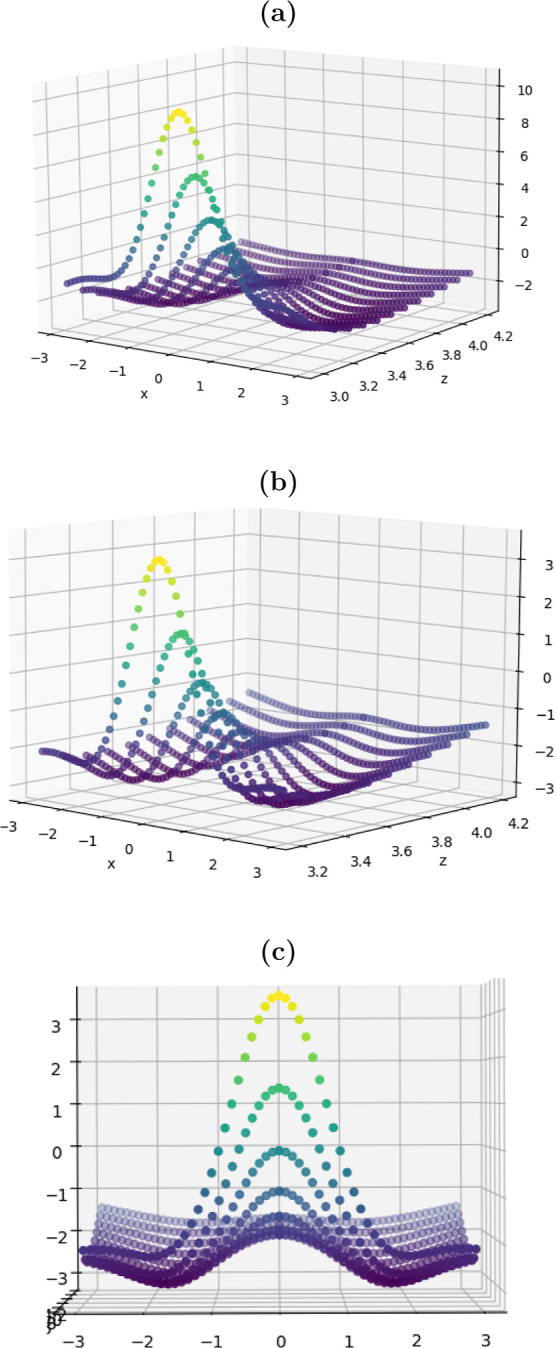
(a) Interaction energy as a function of lateral displacement and vertical separation; (b,c) interaction energy for vertical separations *z* ≥ 3.2 Å.

The energy surface is symmetric with respect to *x* = 0, consistent with the symmetry of identical monomers. For *z* < 3.4 Å, the interaction energy increases sharply near *x* = 0, reflecting strong short-range repulsion. Across all simulated distances, a minimum consistently appears near *x* = 1.7 Å, reproducing the expected equilibrium geometry of the benzene dimer. As visible in Figure 9a, the surface becomes progressively flatter for larger values of *z*, as the π-stacking interaction weakens and eventually vanishes.

Configurations at *z* = 3.0 and *z* = 3.1 Å exhibit extremely large repulsive peaks which dominate the energy scale. To ensure a clearer visualization of the relevant interactions, Figure 9b,c report only the results for *z* ≥ 3.2 Å.

To further explore the geometry of the interaction, translations along the *y*-axis were introduced in addition to *x*. These calculations focused on *x* ∈ [0.0, 2.9] Å, *z* ∈ [3.2, 4.0] Å, and *y* ∈ [0, 1.6] Å. Negative values of *y* were not considered, as they are redundant due to the intrinsic symmetry of the system under investigation. Larger *y* values were also excluded, as this interval is sufficient to reach the equilibrium center–center separation along an alternative carbon–carbon axis, thereby effectively sampling the full range of relevant relative configurations. Finally, larger center–center separations were neglected, as it has been shown previously that the interaction energy progressively vanishes at increasing distances.

A strong correlation emerged between the interaction energy, the vertical separation *z*, and the planar radial distance

$$r_{plan}=\sqrt{x^{2}+y^{2}}$$

Although some scattering appears when translations involve both *x* and *y*, the overall trend remains clearly governed by the radial distance, with configurations at similar *r*_plan_ exhibiting comparable interaction energies.

To make this behavior more evident, Figure 10 presents progressively filtered subsets of the dataset: (a) configurations with *z* > 3.2 Å, which exclude geometries dominated by short-range repulsion; (b) the subset at fixed vertical separation *z* = 3.5 Å, allowing a clearer view of the radial dependence alone; (c) configurations with interaction energy ≤ −2 kcal/mol, corresponding to geometrically favorable π-stacking arrangements.

**Figure 10:**
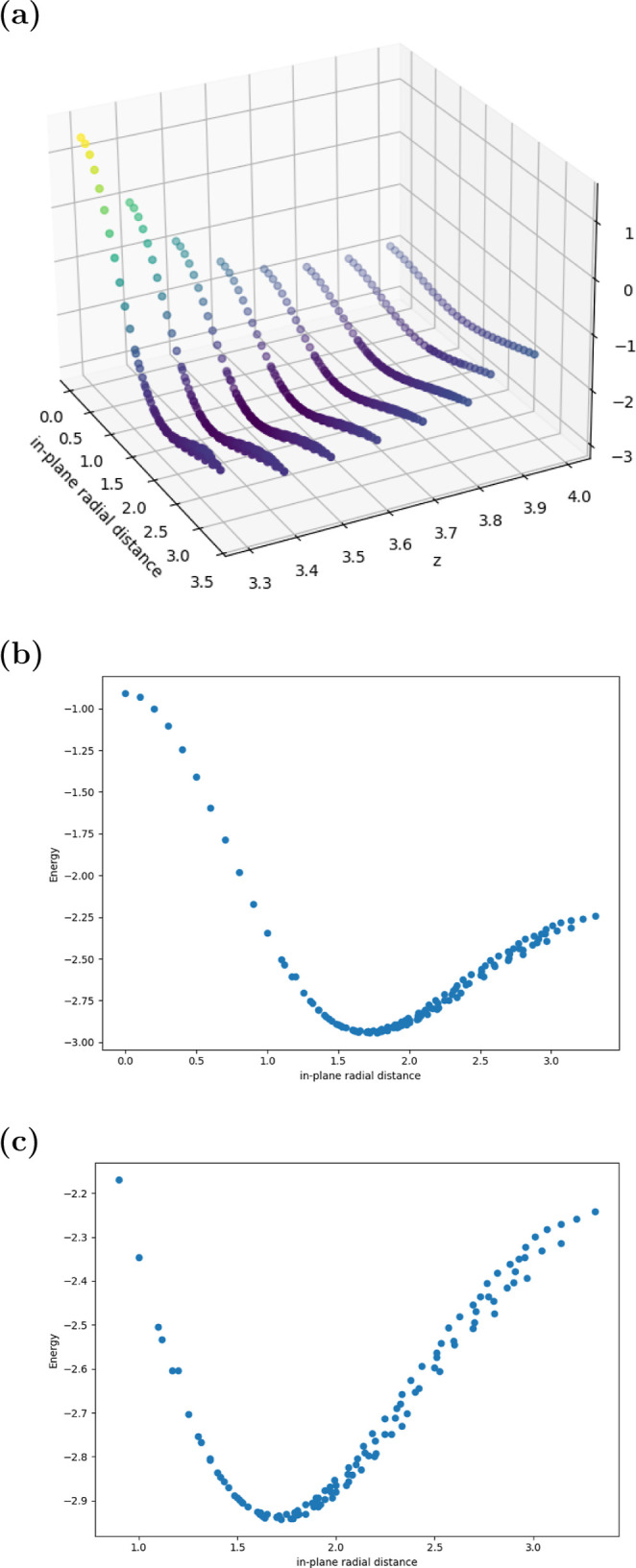
Interaction energy as a function of planar radial distance and vertical separation: (a) *z* > 3.2 Å; (b) fixed *z* = 3.5 Å; (c) *z* = 3.5 Å and energy ≤ −2 kcal/mol.

Near the minimum, values cluster tightly, whereas scattering increases further from the equilibrium configuration. Since the equilibrium benzene dimer energy is approximately −3 kcal/mol, we adopt −2 kcal/mol as a threshold that identifies configurations with significant stabilization. The configurations satisfying this condition are shown in Figure 11a.

**Figure 11:**
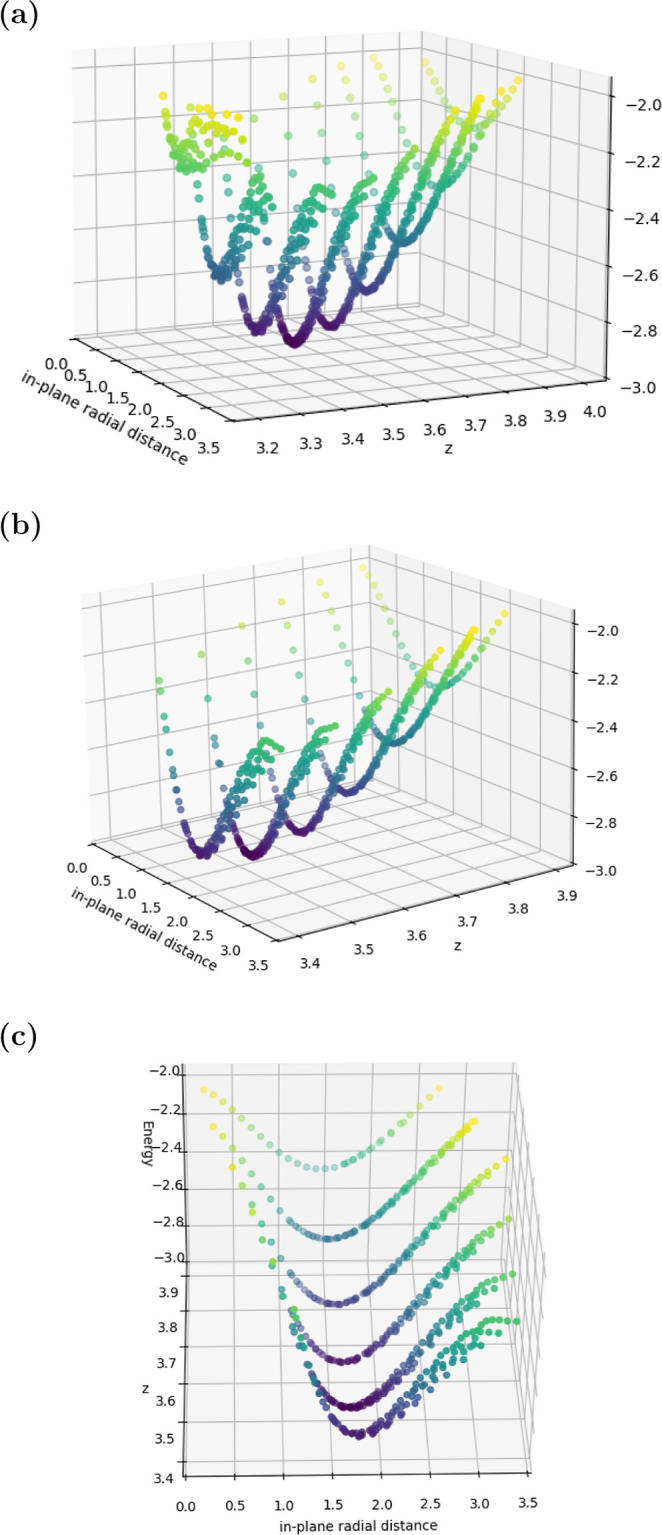
(a) Points with interaction energy ≤ −2 kcal/mol; (b,c) planar radial profiles for *z* ≥ 3.4 Å.

As illustrated in Figure 11a, deviations from the ideal geometry—especially at smaller vertical separations—produce increased fluctuation and irregularity in the energy profile. Conversely, for *z* ≥ 3.4 Å (Figure 11b,c), the dependence on the planar radial distance becomes much more regular.

Finally, we note that different in-plane translations may produce nearly degenerate minima corresponding to symmetric monomer configurations. For example, the configurations (1.7, 0, 3.5) Å and (1.4, 1, 3.5) Å yield energies of −2.944 and −2.942 kcal/mol, respectively. This reflects the fact that the equilibrium geometry corresponds to translation along a carbon–carbon axis, leading to multiple energetically equivalent displacement directions.

Parallel-displaced configuration: rotations after having characterized the translational dependence of the interaction, we extended the analysis to include rotations around each axis of one benzene molecule in order to investigate how changes in relative orientation affect the π-stacking energy. Because aromatic interactions are strongly direction-dependent, this analysis is essential to obtain a complete description of the stacking behavior. As a reference, we adopted the equilibrium parallel-displaced geometry, defined by a lateral displacement of 1.7 Å, a vertical separation of *z* = 3.5 Å, and no translation along the *y*-axis. From this configuration, the molecule centered at the origin was rotated around each of the three principal axes, while the second molecule remained fixed at (1.7, 0.0, 3.5) Å. We first examined rotations within the molecular plane, i.e., around the *z*-axis. Due to the hexagonal symmetry of benzene, only angles between 0° and 30° needed to be considered: a rotation of 60° fully overlaps with the starting geometry, and larger angles are symmetrically equivalent to smaller ones in the opposite direction. The results are shown in Table 1a. In-plane rotation has a negligible effect on the interaction energy: across the sampled angular interval, the energy variation remains below 0.01 kcal/mol. This indicates that rotations within the plane of each molecule do not significantly affect the interaction energy.

**Table 1:** Interaction Energies for the Benzene Dimer in the Parallel-Displaced Configuration^a^

rotation (*z*)	energy (kcal/mol)
(a)	
0°	–2.944
5°	–2.943
10°	–2.942
15°	–2.940
20°	–2.938
25°	–2.937
30°	–2.937

rotation (*x*)	energy(kcal/mol)
(b)	
0°	–2.944
5°	–2.892
10°	–2.722
15°	–2.409

rotation (*y*)	energy(kcal/mol)
(c)	
–15°	–2.258
–10°	–2.666
–5°	–2.880
0°	–2.944
5°	–2.887
10°	–2.738
15°	–2.505

rotation (*x*)	rotation (*y*)	energy(kcal/mol)
(d)		
5°	5°	–2.840
5°	10°	–2.693
5°	15°	–2.460
10°	5°	–2.687
10°	10°	–2.550
10°	15°	–2.317
15°	5°	–2.408
15°	10°	–2.288
15°	15°	–2.063

a The reference geometry has a lateral displacement along the *x*-axis of 1.7 Å, no translation along the *y*-axis, and a vertical separation of *z* = 3.5 Å. Rotations considered are (a) in-plane rotation, (b) rotation around the *x*-axis, (c) rotation around the *y*-axis, (d) combined *x*- and *y*-axis rotations.

We then investigated rotations around the *x*- and *y*-axes, which tilt the aromatic ring out of plane and therefore disrupt the parallel alignment essential for π-stacking. A preliminary exploration with angles up to 60° showed that even moderate tilting dramatically weakens the interaction. To focus on the energetically relevant region, we restricted the analysis to angles between 0° and 15°, and to [−15°, 15°] for the asymmetric rotation around the *y*-axis. Table 1b,c report the corresponding interaction energies. Even small tilting angles lead to a significant loss of stabilization, confirming that the parallel orientation is a critical determinant of π-stacking stability. The effect is more pronounced for rotations around the *x*-axis, but rotations around the *y*-axis also lead to rapid destabilization, with clear asymmetry due to the nonequivalent geometry of the displaced molecule. Finally, Table 1d presents the combined effect of simultaneous rotations around both axes. The interaction energy decreases even more rapidly under these combined distortions, illustrating how deviations from coplanarity along multiple directions strongly suppress the magnitude of the stacking interaction.

Thanks to the rotational calculations, we can clearly understand why the interaction energy does not depend independently on the *x*- and *y*-displacements. Since in-plane rotations were found to have only a minimal effect on the total interaction energy, a translation along the *y*-axis and the *x*-axis is effectively equivalent to a translation along the *x*-axis followed by such a rotation. Therefore, the system cannot distinguish between these two directions, and the interaction energy naturally depends mainly on the radial in-plane distance rather than on the individual Cartesian components.

### T-Shaped Configuration: Translations and Rotations

We analyze the interaction energy of the benzene dimer in the T-shaped configuration, starting from the equilibrium geometry in which one molecule lies on the *xy*-plane, centered at the origin, while the second molecule is oriented perpendicularly, with its center located at (0, 0, 5). A schematic representation of the reference configuration is shown in Figure 2.

Starting from this geometry, the vertically oriented molecule was translated along the three Cartesian axes in order to investigate the dependence of the interaction energy on relative displacements. The equilibrium interaction energy in the T-shaped configuration is found to be slightly more favorable than in the parallel-displaced case, with a minimum value of −3.101 kcal/mol compared to −2.944 kcal/mol. Although the difference is modest, it indicates a marginally higher stability of the T-shaped arrangement within the present computational framework.

The interaction energy as a function of translations along the Cartesian directions is discussed with reference to Figure 12a,b. The results show a stronger sensitivity to lateral displacements along the *y*-axis than along the *x*-axis, particularly at short vertical distances. This behavior reflects the geometric asymmetry of the T-shaped configuration, which makes the lateral component along *y* more relevant for electronic overlap between the two molecules. As done previously for the parallel-displaced configuration, in Figure 13 we report the interaction energy as a function of the vertical distance *z* and the planar radial distance between the centers of the two molecules. In this representation, only energy values less than or equal to −2 kcal/mol are considered, in order to more clearly highlight the energetically significant configurations and avoid introducing excessive data dispersion.

**Figure 12:**
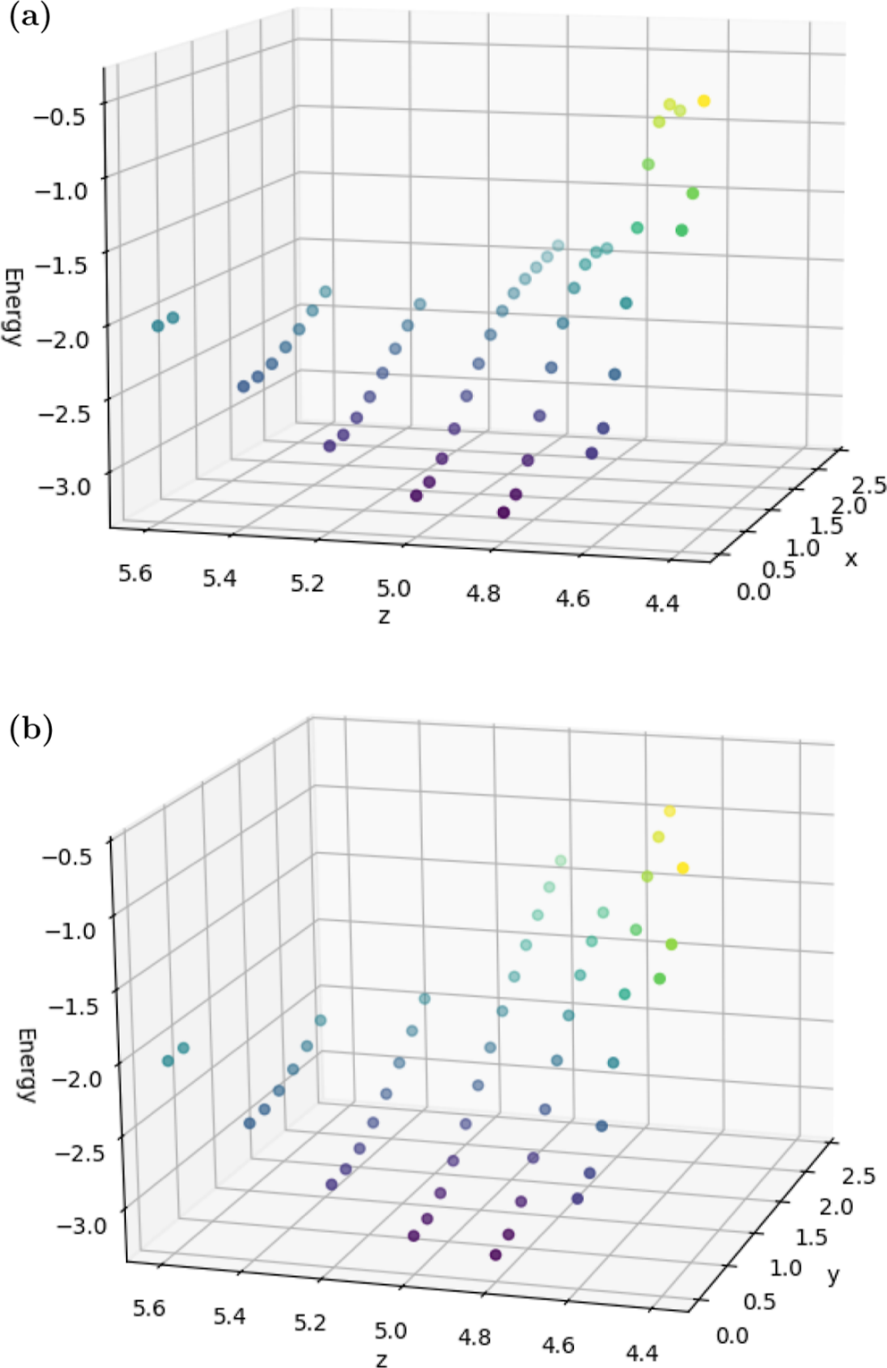
Interaction energy as a function of translation along: (a) *x* and *z*; (b) *y* and *z*.

**Figure 13:**
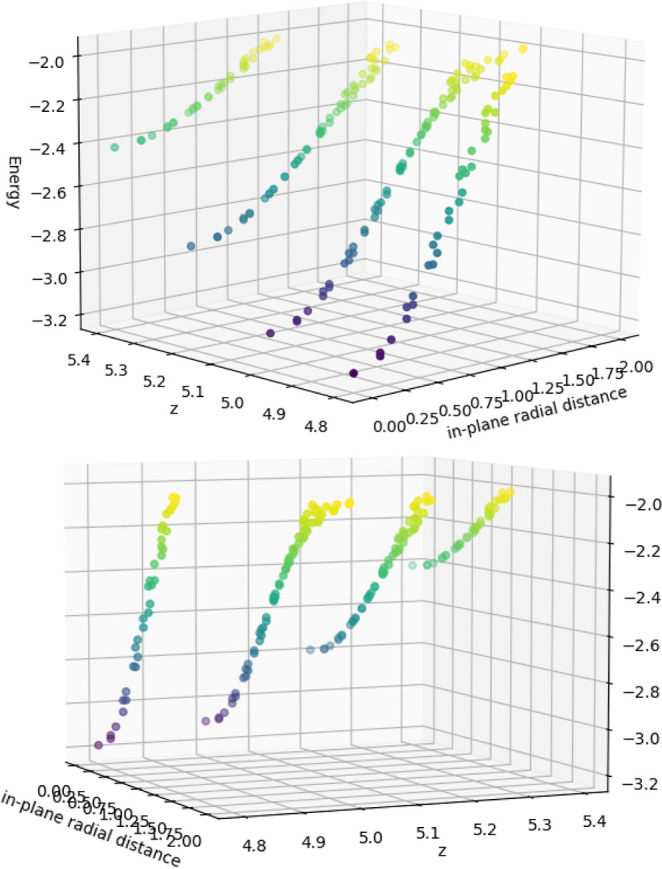
Interaction energy as a function of the vertical distance *z* and the planar radial distance, with energy ≤ −2 kcal/mol.

Rotational effects were analyzed by rotating the vertically oriented molecule around the Cartesian axes. The interaction energy is essentially invariant under rotations around the *z*-axis, as shown in Table 2a, indicating a high degree of rotational symmetry around the molecular axis. Small variations observed in this case are attributable to numerical noise rather than to genuine physical effects.

**Table 2:** Interaction Energies for Rotations in the T-Shaped Configuration of the Benzene Dimer^a^

rotation (*z*)	energy (kcal/mol)
(a)	
0°	–3.101
5°	–3.103
10°	–3.103
15°	–3.103
20°	–3.102
25°	–3.102
30°	–3.102

rotation (*x*)	energy(kcal/mol)
(b)	
0°	–3.101
5°	–3.072
10°	–2.980
15°	–2.827

rotation (*y*)	energy(kcal/mol)
(c)	
0°	–3.101
5°	–3.081
10°	–3.025
15°	–2.940

rotation (*x*)	rotation (*y*)	energy(kcal/mol)
(d)		
5°	5°	–3.052
5°	10°	–2.998
5°	15°	–2.918
10°	5°	–2.963
10°	10°	–2.917
10°	15°	–2.854
15°	5°	–2.814
15°	10°	–2.785
15°	15°	–2.742

a The reference geometry has a lateral displacement along the *x*-axis of 0 Å, no translation along the *y*-axis, and a vertical separation of *z* = 5 Å. Rotations considered are (a) rotations around the *z*-axis, (b) rotations around the *x*-axis, (c) rotations around the *y*-axis, (d) combined *x*–*y* rotations.

Rotations around the *x*- and *y*-axes lead to a gradual weakening of the interaction, as reported in Table 2b,c, respectively. However, even for rotations up to 15°, the interaction remains significantly attractive, especially for rotations around the *y*-axis. Combined rotations around both axes, summarized in Table 2d, confirm the overall robustness of the T-shaped configuration with respect to orientational perturbations.

Overall, we observe that the T-shaped configuration is significantly less sensitive to rotations compared to the parallel-displaced configuration. In fact, even with 15° rotations, the intensity of the interaction does not decrease significantly, especially with respect to rotations around the *y*-axis.

### Mathematical Modeling

Our goal is to integrate the π-stacking interaction into the physical Hamiltonian introduced in the introduction. This integration presents a specific conceptual challenge: incorporating an interaction that is intrinsically molecular into a computational framework whose basic elements are individual atoms. The graph representation used for both the protein and the ligand naturally encodes atomic positions but does not, in its original form, provide an intuitive mechanism for expressing interactions that depend on the collective geometry of entire aromatic fragments. The limitation also arises from the structure of the QUBO model itself. Since the optimization variables control the placement of pairs of atoms at a time, the model can directly enforce geometric constraints only on atom pairs. However, determining the spatial orientation of a benzene ring requires fixing at least three noncollinear atoms. This mismatch between the atomic granularity of the QUBO formulation and the molecular nature of the π-stacking interaction makes a direct encoding impossible without introducing additional modeling layers.

For this reason, an approximation becomes necessary. Fortunately, our quantum-chemical calculations reveal a key physical property: rotations of a benzene molecule within its own plane produce only negligible variations in the interaction energy compared to the total stabilization. This observation suggests that, rather than attempting to represent all rotational degrees of freedom of the aromatic ring, it is sufficient—and physically justified—to retain only the orientation of the normal vector to the plane of the fragment. By discarding in-plane rotational information, we obtain a reduced yet accurate and computationally tractable description that preserves the essential physics of π-stacking interaction while remaining compatible with the discrete structure of the QUBO formulation.

To encode the orientation of each aromatic fragment within a discrete framework, we introduce two additional nodes for every aromatic ring of the ligand in the molecular graph *G*_mol_: one representing the center of the ring and a second auxiliary vertex. The auxiliary node is placed along the direction of the normal vector to the plane of the ring, so that the oriented segment connecting the ring center to the auxiliary vertex uniquely encodes the orientation of the aromatic fragment. Figure 3a illustrates this construction.

We then enrich the labeling system of the molecular graph by introducing, for each node *i*.

- A binary variable ${ĉ}_{i}$ , equal to 1 if *i* is the center of a benzenic fragment, and 0 otherwise;
- A binary variable *c*_*ij*_, equal to 1 if node *i* is the additional vertex defining, together with its center *j*, the normal direction to the aromatic plane.

This yields the augmented molecular graph

$$G_{mol}=\left\{i,e_{ij},w_{ij},q_{i},{ĉ}_{i},c_{ij}\right\}$$

To determine where a ligand aromatic fragment can be positioned in space, we consider a subgraph of *G*_grid_, denoted *G*_center_, whose vertices represent spatial locations compatible with a favorable π-stacking interaction. For each such vertex *i*′, we define a set of admissible orientation vertices

$$\left\{j_{1}^{\prime },j_{2}^{\prime },...,j_{n}^{\prime }\right\}\subseteq G_{grid}$$

such that the segment *i* ^′^ *j*_*k*_^′^ forms an angle with the protein aromatic plane lying in the interval

$$\left[\frac{\pi }{2}-\alpha ,\frac{\pi }{2}+\alpha \right]$$

where the tolerance parameter α > 0 controls the deviation from perfect parallelism between the two aromatic planes.

Figure 3b,c show an example of this construction: the center of ligand’s benzenic fragment (in green) lies in *G*_center_, and its auxiliary vertex falls within a spherical surface corresponding to the admissible orientations.

We therefore introduce, on the grid graph *G*_grid_, the following labels:

- A variable ${ĉ}_{i^{\prime }}=1$ if *i*′ is a possible favorable position for the ligand aromatic ring center, i.e. if *i*′ ∈ *G*_center_;
- A continuous variable *c*_*j*′*i*′_, encoding the geometric and energetic quality of the orientation defined by the segment *i*′*j*′.

The value of *c*_*j*′*i*′_ is obtained as follows. For each admissible orientation *i*′ → *j*′, the interaction energy between the ligand’s benzenic fragment and the protein’s aromatic ring is computed using the quantum-chemical calculations described earlier. Fixing the direction of the fragment normal, identified with the oriented segment *i*′ → *j*′, an energy interval is obtained by rotating the aromatic ring around the *i*′*j*′ axis, which coincides with its normal axis. Since the proposed discrete model does not explicitly account for this rotational degree of freedom, an effective energetic descriptor is introduced by assigning to *c*_*j*′*i*′_ the average value of the corresponding energy interval. If *j*′ does not belong to the admissible orientation set, we simply set *c*_*j*′*i*′_ = 0.

This approach yields a flexible representation of spatial alignment: rather than enforcing a rigid accept/reject rule, the model assigns an energy contribution proportional to the degree of geometric compatibility.

The enriched grid structure becomes

$$G_{grid}=\left\{i^{\prime },e_{i^{\prime }j^{\prime }},w_{i^{\prime }j^{\prime }},V_{i^{\prime }},{ĉ}_{i^{\prime }},c_{i^{\prime }j^{\prime }}\right\}$$

The Hamiltonian term encoding the π-stacking interaction in the parallel-displaced configuration is therefore defined as

1 $$H_{\pi }=\sum_{i,i^{\prime }}{ĉ}_{i}{ĉ}_{i^{\prime }}x_{ii^{\prime }}\left(\sum_{j,j^{\prime }}c_{ij}c_{i^{\prime }j^{\prime }}x_{jj^{\prime }}\right)$$

### Extension to the T-Shaped Configuration

Calculations performed for the T-shaped arrangement of the benzene dimer reveal a complementary behavior. When one ring is oriented nearly perpendicular to the other, rotations of the horizontal fragment around its own normal axis (i.e., the axis orthogonal to its plane) induce only minor variations in the interaction energy. This suggests that, similarly to the parallel-displaced case, the interaction can be effectively modeled by fixing the axis of the vertical fragment, while leaving rotations around this axis unconstrained.

Motivated by this result, we introduce for each T-shaped center *i* ^′^∈*G*_center_^*T*^ a set of nodes

$$\left\{j_{1}^{\prime },j_{2}^{\prime },...,j_{n}^{\prime }\right\}\subseteq G_{grid}$$

representing positions where one carbon atom of the ligand aromatic ring may lie when the fragment is properly oriented in a T-shaped arrangement relative to the protein’s benzene ring. A corresponding visualization is provided in Figure 4.

As before, we define a continuous label *c*_*i*′*j*′_ quantifying the interaction quality associated with the orientation *i*′ → *j*′. The Hamiltonian associated with the T-shaped configuration then takes the form

$$H_{\pi ,T‐shaped}=\sum_{i,i^{\prime }}{ĉ}_{i}{ĉ}_{i^{\prime }}x_{ii^{\prime }}\left(\sum_{j,j^{\prime }}c_{ij}c_{i^{\prime }j^{\prime }}x_{jj^{\prime }}\right)$$

with the variables interpreted as follows: ${ĉ}_{i}=1$ if node *i* is the center of a ligand aromatic fragment; ${ĉ}_{i^{\prime }}=1$ if node *i*′ is a T-shaped candidate center in the grid graph; *c*_*ij*_ = 1 if node *j* is a carbon atom belonging to the aromatic fragment centered at *i*; *c*_*i*′*j*′_ is a continuous scalar representing the interaction quality for that specific orientation.

This formulation provides a coherent and physically grounded mechanism for modeling both parallel-displaced and T-shaped π-stacking configurations within a unified QUBO framework. By encoding orientations through additional vertices and labels, the model effectively captures the geometric subtleties revealed by the calculations while maintaining compatibility with discrete optimization methods.

### Simulated Annealing Calculations

We now perform simulated annealing calculations to minimize the Hamiltonian introduced in the Introduction, now augmented by the additional term to account for the π–π stacking interactions. In this section, we report only the annealing results corresponding to the parallel-displaced configuration, as the computations for the T-shaped configuration follow an entirely analogous procedure and lead to qualitatively similar outcomes. The optimization experiments were carried out using the classical simulated annealing algorithm implemented in neal.SimulatedAnnealingSampler (v. 1.1.3). The formulation has been explicitly designed to be compatible with quantum annealing architectures, and its deployment on D-Wave quantum platforms is therefore a natural extension of the present study, which we reserve for future work.

To assess the behavior of the proposed model, we first performed simulated annealing calculations on small test systems. Working with simple aromatic fragments allows for a direct comparison between the predicted configurations and the interaction patterns expected from quantum-chemical calculations, without the complexity introduced by full protein environments.

In typical docking applications, one considers protein pockets containing hundreds of atoms surrounding the binding site. In such settings, disentangling the individual contributions of different interaction mechanisms becomes nontrivial. A key role is played by the multiplicative parameters λ introduced in the General Structure of the Hamiltonian Subsection, which rescale the various energy terms so that they become comparable in magnitude. For instance, the geometric term in the Hamiltonian can easily reach values of order 10^1^ to 10^2^, especially when the grid points do not perfectly coincide with ligand’s atomic positions, whereas the residual term introduced in this work typically ranges between −7 and 0. Without an appropriate λ-scaling, the latent contribution would be effectively negligible when compared with the dominant geometric or van der Waals terms. Determining suitable λ-values is therefore essential for correctly incorporating the π-stacking term into the QUBO formulation. A comprehensive calibration of the λ-parameters is beyond the scope of this work and will be the subject of future studies. Here, we focus on demonstrating that the newly introduced residual term is able to guide the system toward physically meaningful configurations in a series of controlled test cases.

### QUBO Coefficient

The Hamiltonian parameters are derived from quantum-chemical calculations, as detailed in the Quantum-Chemical Calculations Results section, which provide the total interaction energy between two benzene rings. Since the Hamiltonian presented in the introduction already includes explicit Coulomb and van der Waals contributions, these components must be removed from the calculation data to avoid double counting. We therefore subtract the analytically computed electrostatic and van der Waals terms from the total interaction, thus obtaining

$$E_{residual}=E_{tot}-\left(E_{Coulomb}+E_{VdW}\right)$$

The residual contribution, also referred as latent energy, accounts for collective effects, including those associated with π-stacking interactions.

Only configurations with interaction energies below −2 kcal/mol are retained, ensuring that the data set includes only geometries where stacked configurations can be observed at room temperature and excluding the repulsive peaks observed at zero displacement (Figure 9a). For these configurations, subtracting the electrostatic and van der Waals contributions yields the latent energies used to determine the QUBO coefficients for the residual component of the Hamiltonian.

The data points obtained were then fitted using a third-degree polynomial in the variables *d* (displacement) and *z* (interplanar distance). The resulting polynomial model is given by

$$\begin{array}{l} f\left(d,z\right)=-0.413d^{3}+12.322z^{3}-0.730d^{2}z+4.638dz^{2}+5.341d^{2}\\ -150.773z^{2}-33.423dz+55.572d+613.092z-827.530 \end{array}$$

The goodness of fit is confirmed by the coefficient of determination, which is *R* ^2^ = 0.998.

Since the modeling approach presented does not explicitly resolve rotations of the molecules around the *z*-axis, i.e. around the axis normal to the benzenic plane, the interaction energy is modeled primarily as a function of the lateral displacement and the vertical separation between the molecular planes. Although such rotations are not treated as explicit degrees of freedom in the Hamiltonian, the energetic variability associated with them is implicitly taken into account by averaging the interaction energies over the corresponding rotational configurations, as discussed in detail below.

In this framework, we deliberately avoid expressing the interaction energy as a function of the planar radial distance. Indeed, variations along the *y* coordinate effectively combine changes in the interplanar separation with rotations around the *z*-axis, thereby introducing rotational effects that are not explicitly represented in the Hamiltonian. Restricting the analysis to configurations parametrized solely by vertical and lateral displacements ensures internal consistency with the assumptions underlying the adopted theoretical model.

Due to the high computational cost of sampling rotational degrees of freedom, rotational effects were explicitly computed only at the equilibrium configuration. From these calculations, the relative variation in interaction energy induced by rotations was estimated. Assuming that this variation remains approximately constant across the explored configurational space, rotational effects were incorporated through a multiplicative correction applied to the translational interaction energy. This approximation enables an efficient inclusion of rotational contributions while significantly reducing the number of required calculations.

In particular, the percentage variations in interaction energy associated with rotations around the *x*-axis, corresponding to the direction of lateral displacement, are parametrized by the angle α, while those associated with rotations around the *y*-axis are parametrized by the angle β. Due to the symmetry of the molecular configuration with respect to positive and negative rotations around the displacement axis, the values of α are restricted to positive angles, whereas both positive and negative values of β are allowed, as no analogous symmetry constraint applies to rotations around the *y*-axis. For each pair (α, β), the reported values are obtained by averaging the interaction energies over the corresponding energetic intervals, which also account for rotations around the axis normal to the benzenic fragment, as described in the Mathematical Modeling Section.

These data are interpolated by the polynomial function

$$\begin{array}{l} g\left(\alpha ,\beta \right)=-8.95\times 10^{-6}\alpha^{3}+1.57\times 10^{-5}\beta^{3}+1.34\times 10^{-5}\alpha^{2}\beta -2.54\times 10^{-6}\alpha \beta^{2}\\ -6.71\times 10^{-4}\alpha^{2}-9.15\times 10^{-4}\beta^{2}-4.79\times 10^{-5}\alpha \beta \\ +6.64\times 10^{-4}\alpha +5.15\times 10^{-4}\beta +1.00 \end{array}$$

with *R* ^2^ = 0.999.

In conclusion, the coefficients *c*_*i*′*j*′_ introduced in eq 1 are determined through the following procedure:

- Benzene fragments sufficiently close to the binding pocket are identified, and their centroids and aromatic plane normals are computed.
- For each fragment, grid points *P* are selected such that the vertical distance from the aromatic plane satisfies *z* ∈ [3.2, 4.0] Å and the lateral displacement lies in *d* ∈ [1.0, 3.0] Å.
- Suitable anchor points *V* are identified within a distance of 1–2 Å from each *P*, such that the angles α and β formed with the displacement axis and its orthogonal direction do not exceed 15°.
- The coefficient *c*_*i*′*j*′_ is finally assigned as

$$c_{i^{\prime }j^{\prime }}=f\left(d,z\right)g\left(\alpha ,\beta \right)$$

### Ligand and Pockets

As a first validation step, we consider the interaction between two benzene rings, which serves as a minimal reference system. We then extend the analysis to the interaction between a benzene ligand and three aromatic amino acids—tyrosine, phenylalanine, and tryptophan—shown in Figure 5. In these cases, the full amino acid structures are included in the calculations, rather than considering only their isolated aromatic moieties, so as to account for the influence of the surrounding molecular framework on the interaction. These residues are among the most frequently involved in π–π interactions, as highlighted, for example, by Shao et al.^29^ and Burley and Petsko.^30^ We first analyzed the interaction with tyrosine (C_9_H_11_NO_3_, Figure 5a), which contains a phenol group with a hydroxyl substituent in the para position on the aromatic ring. For comparison, we also considered phenylalanine (C_9_H_11_NO_2_, Figure 5b), featuring an unsubstituted phenyl ring that is purely hydrophobic and capable of participating in π-stacking interactions. Finally, we analyzed tryptophan (C_11_H_12_N_2_O, Figure 5c), which contains an indole group - a benzene ring fused to a pyrrole - forming a larger conjugated aromatic system.

In our calculations, tyrosine, phenylalanine, and tryptophan are modeled as complete amino acid residues, including both the full peptide backbone (N, CA, C, and O atoms) and the aromatic side chain. No truncation of the backbone is applied. All systems are considered in their standard protonation state under physiological pH conditions, corresponding to chemically reasonable neutral forms for the residues investigated in this work.

Heterocyclic amino acids like histidine and tryptophan are classified as aromatic because their side-chain rings satisfy Hückel’s rule (4*n* + 2 π electrons, planarity, and continuous conjugation), even though they contain noncarbon atoms (nitrogen) in the ring. However, Histidine is frequently excluded from the core group of “aromatic amino acids” (phenylalanine, tyrosine, and tryptophan) primarily due to its chemical behavior rather than its structure. For example, although the imidazole ring in histidine is aromatic, its classification is primarily driven by its basic character, as its p*K*_*a*_ (≈6.0) lies close to physiological pH. This allows histidine to readily switch between a neutral and a positively charged (protonated) state, introducing an additional level of complexity and variability that is not present in purely hydrophobic aromatic residues such as phenylalanine, tyrosine, and tryptophan.

Furthermore, although histidine can participate in π–π stacking interactions, its ability to form hydrogen bonds owing to its heteroatoms and potential charge distinguishes its behavior from other aromatic residues.

For these reasons, including histidine would require a more detailed treatment of protonation equilibria and electrostatic effects, which falls beyond the scope of the present modeling framework. As a consequence, it has been excluded from simulated annealing calculations in the present study.

The ligand, presented in Figure 6is a benzene molecule enriched with two auxiliary nodes: the aromatic center and a vertical anchoring node placed 1.5 Å above the ring center along the normal direction. These nodes are fictitious–they do not correspond to physical atoms and therefore carry no partial charges or van der Waals parameters.

### Pocket Grids

To rigorously test the model, we designed three synthetic pocket grids centered around the aromatic pocket fragment, which are presented in Figure 7. For clarity, the green nodes represent the reference case in which the pocket is defined by a single benzene molecule, while the blue nodes correspond to the three different pocket grids. The benzene ring in the protein does contain hydrogen atoms, but to improve visualization and avoid an overly crowded and confusing image, the hydrogens have been omitted. In all three grids, the theoretically predicted equilibrium position of the ligand is indicated by dashed lines.

- Grid A (Figure 7a): a minimal grid containing only the nodes required to place the ligand in a single ideal pose. It consists of 14 nodes: 6 carbon positions, 6 hydrogen positions, 1 aromatic center, and 1 vertical anchoring node. The center node is placed at a lateral displacement of approximately 1.7 Å and at an interplanar distance of about 3.4 Å, with the segment joining the center and the vertical node aligned with the normal to the pocket’s aromatic plane.
- Grid B (Figure 7b): obtained by enriching Grid A with 26 additional nodes placed in its vicinity, including a complete second copy of the original 14-node ligand-compatible arrangement. This introduces an additional allowed candidate pose and increases spatial flexibility for the ligand.
- Grid C (Figure 7c): to further expand the search space and evaluate the robustness of the model under more complex conditions, a third set of 14 nodes was introduced, representing an additional geometrically favorable configuration for the benzene fragment. This setup enables the assessment of the model’s ability to consistently identify optimal binding poses in the presence of multiple competing interaction sites.

The pocket-grid points are arranged so as to reproduce geometries compatible with favorable π-stacking interactions relative to the aromatic side chain of each amino acid, namely the phenyl ring in phenylalanine and tyrosine and the indole ring in tryptophan. In this configuration, the interaction is primarily localized between the benzene molecule and the aromatic moiety of the side chain, whereas the peptide backbone remains spatially separated from the interaction region. Consequently, the backbone does not directly participate in the π–π interaction, although it retains an important structural role by constraining the spatial arrangement of the amino acid with respect to the pocket grid.

To further clarify the geometry of the modeled systems, representative structures of the amino acid-benzene complexes are reported in Figure 14. The figure explicitly includes the complete amino acid backbone and highlights how the amino acid is positioned relative to the pocket-grid structure, where for simplicity and clarity only Grid A is shown.

**Figure 14:**
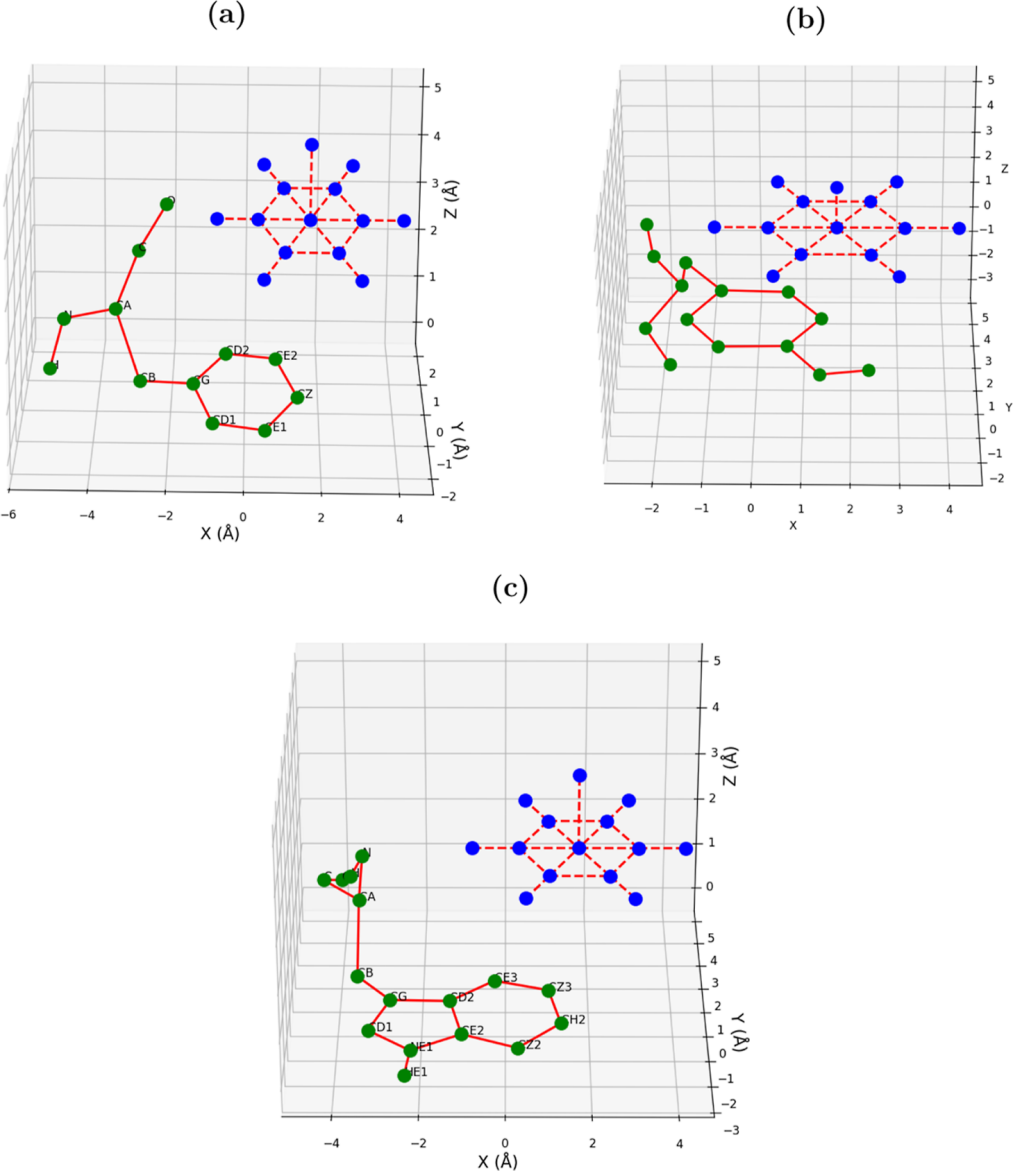
Representative π-stacking conformations for the three amino acid-pocket-grid systems. (a) Phenylalanine system; (b) tyrosine system; (c) tryptophan system. Each panel shows the complete amino acid residue (backbone and side chain) together with the aromatic ring embedded in the pocket grid, positioned in a configuration compatible with π-stacking interactions with the side chain.

### Results and Discussion

In all tests, the Hamiltonian included geometric, electrostatic, van der Waals, and residual contributions, while hydrogen-bonding and hydrophobic terms were neglected. Although hydrogen-bonding and hydrophobic contributions were included in the original formulation of the Hybrid Hamiltonian model described by Micucci et al.,^15^ in the present work we chose not to subtract these terms from the interaction energies obtained from quantum chemistry calculations. In particular, hydrogen bonding is not relevant in the systems considered here, since we are dealing with benzene molecules, and carbon atoms do not possess sufficient electronegativity to participate in hydrogen-bond interactions. Therefore, this contribution is not expected to play any meaningful role in the interaction energy. Regarding hydrophobic interactions, in the Hybrid Hamiltonian formulation^15^ they were not modeled through an explicit physical energy term, but rather through a heuristic counting scheme assigning contributions based on atom pairs satisfying certain structural criteria. Since our interaction energies are obtained from quantum chemistry calculations and subsequently decomposed by explicitly subtracting well-defined van der Waals and electrostatic contributions, we did not include the subtraction of hydrophobic contributions, as these are not consistently defined within the same physically grounded energy decomposition framework. Therefore, when the van der Waals and Coulomb energies are subtracted from the total one, the difference implicitly accounts for (although not in an explicit way) hydrophobic interactions.

The indexing of the ligand nodes is shown in Figure 6. For the pocket grids, nodes are indexed such that nodes 0–5 correspond to the carbon atom positions, nodes 6–7 represent the aromatic center and the vertical anchor, and nodes 8–13 denote the hydrogen atoms in the theoretically predicted equilibrium configuration.

The rows labeled “selected couplers” in Tables 4 to 14 list the ordered pairs of nodes selected by the annealing procedure, indicating how ligand nodes are mapped onto pocket grid nodes. For example, the pair (12,6) means that ligand node 12 is mapped to node 6 of the grid.

#### Benzene–Benzene

We first considered a benzene–benzene system, using only the aromatic fragment of the pocket molecule. For Grid A, a minimal configuration tightly matching the ligand geometry, the simulated annealing results are summarized in Table 3. The chosen Lambda Weights (λ_geom_, λ_el_, λ_vdw_, λ_residual_), number of reads, and sweeps are reported in the table. We remark that the parameters λ_hydrogen_ and λ_hydrophobic_, introduced in the original formulation proposed by Micucci et al.,^15^ are not reported here, as they have been set identically equal to zero, for the reasons already discussed at the beginning of the Results and Discussion section. It is important to observe that since the different terms entering the formulation have heterogeneous physical nature and scale, the resulting Hamiltonian *H* is not intended to represent a physical energy, but rather an optimization objective in arbitrary units. Accordingly, the λ coefficients should be interpreted as tunable weighting factors rather than physical coupling constants.

**Table 3:** Results of the Simulated Annealing Computation Performed Using a Benzene Ligand, a Benzene Molecule, and Pocket Grid A^a^

Configuration	
lambda weights	[1.0, 1.0, 1.0, 1.0]
number of reads	100
number of sweeps	2000
Problem Size	
nonzero linear terms	196
nonzero quadratic terms	11 101
total nonzero terms	11 297
selected couplers	{(9,13),(0,3),(10,9),(11,8),(12,6),(13,7),(1,4)
	(2,2),(7,12),(3,5),(4,1),(5,6),(6,11),(8,10)}
Energies	
best energy	–3.394
geometric energy	0.003
vdW energy	–2.315
electrostatic energy	1.582
residual energy	–2.664

a Execution time: 1.49 s.

The optimal solution correctly maps the ligand atoms onto the intended grid positions, with a total energy of about −3.39. The geometric component, which models a displacement mismatch penalty, is essentially zero, indicating a nearly perfect match between ligand and pocket geometry, while the van der Waals and residual terms provide stabilizing contributions of similar magnitude to each other. The electrostatic term is slightly positive but does not offset the overall stabilization. The sum of van der Waals, electrostatic, and residual terms is close to −3, in agreement with the reference calculations.

When the same ligand–pocket pair is embedded in the larger Grid B, the results in Table 4 show that the optimal configuration remains essentially unchanged, apart from a rotation around the center–anchor axis, which is not penalized by the geometric term within the complete Hamiltonian. The contributions are virtually identical, confirming the robustness of the model under moderate grid enrichment.

**Table 4:** Results of the Simulated Annealing Computation Performed Using a Benzene Ligand, a Benzene Molecule, and Pocket Grid B^a^

Configuration	
lambda weights	[1.0, 1.0, 1.0, 1.0]
number of reads	500
number of sweeps	10000
Problem Size	
nonzero linear terms	574
nonzero quadratic terms	92 289
total nonzero terms	92 863
selected couplers	{(0, 1), (5, 4), (10, 11), (11, 12), (4, 3), (13, 7), (1, 0)}
	{(12, 6), (2, 2), (6, 9), (7, 8), (3, 5), (8, 10), (9, 13)}
Energies	
best energy	–3.389
geometric energy	0.008
vdW Energy	–2.315
electrostatic energy	1.582
residual energy	–2.664

a Execution time: 11.19 s.

For Grid C, the problem size and energy landscape become more complex. Using the same annealing parameters, the algorithm fails to recover the exact optimal mapping, as shown in Table 5. Nevertheless, the aromatic center and vertical node are still placed correctly, so that the residual term remains nonzero. Increasing the weight of the geometric term (e.g., setting λ_geom_ = 3) restores the correct mapping, demonstrating that the λ-parameters can be used to enforce structural fidelity. The ligand placement within Grid C for the case λ_geom_ = 3 is illustrated in Figure 8.

**Table 5:** Results of the Simulated Annealing Computation Performed Using a Benzene Ligand, a Benzene Molecule, and Pocket Grid C^a^

Configuration	
lambda weights	[1.0, 1.0, 1.0, 1.0]
number of reads	500
number of sweeps	10000
Problem Size	
nonzero linear terms	770
nonzero quadratic terms	165 382
total nonzero terms	166 152
selected couplers	{(0, 4), (2, 3), (11, 9), (10, 44), (4, 2), (1, 5), (3, 0)}
	{(12, 6), (13, 7), (8, 11), (5, 1), (6, 12), (7, 37), (9, 8)}
Energies	
best energy	–1.387
geometric energy	2.549
vdW energy	–2.424
electrostatic energy	1.152
residual energy	–2.664

a Execution time: 24.77 s

### Tyrosine-Benzene

We then replaced the pocket benzene ring with a tyrosine fragment. On Grid B, the results reported in Table 6 show that the ligand again adopts a configuration that activates the residual term, with a total energy lower than in the benzene–benzene case due to the additional van der Waals contributions from the tyrosine side chain.

**Table 6:** Results of the Simulated Annealing Computation Performed Using a Benzene Ligand, a Tyrosine Molecule, and Pocket Grid B^a^

Configuration	
lambda weights	[1.0, 1.0, 1.0, 1.0]
number of reads	500
number of sweeps	10000
Problem Size	
nonzero linear terms	574
nonzero quadratic terms	92 289
total nonzero terms	92 863
selected couplers	{(11,8),(0,3),(4,1),(1,4),(5,0),(13,7),(12,6),(2,2)
	(7,12),(3,5),(10,9),(6,11),(8,10),(9,13)}
Energies	
best energy	–4.633
geometric energy	0.003
vdW energy	–3.057
electrostatic energy	1.086
residual energy	–2.664

a Execution time: 11.40 s.

On Grid C, the increased number of nodes once more makes it harder for simulated annealing to identify the global minimum with fixed parameters (Table 7). However, by increasing λ_geom_ to 3.0, the correct minimum-energy configuration is recovered (Table 8).

**Table 7:** Results of the Simulated Annealing Computation Performed Using a Benzene Ligand, a Tyrosine Molecule, and Pocket Grid C^a^

Configuration	
lambda weights	[1.0, 1.0, 1.0, 1.0]
number of reads	500
number of sweeps	10000
Problem Size	
nonzero linear terms	770
nonzero quadratic terms	165 382
total nonzero terms	166 152
selected couplers	{(0, 3), (11, 41), (1, 4), (12, 6), (13, 7), (10, 9), (2, 2)}
	{(3, 5), (7, 12), (4, 1), (5, 0), (6, 11), (8, 10), (9, 21)}
Energies	
best energy	–4.018
geometric energy	–1.278
vdW energy	–3.156
electrostatic energy	0.524
residual energy	–2.664

a Execution time: 25.13 s

**Table 8:** Results of the Simulated Annealing Computation Performed Using a Benzene Ligand, a Tyrosine Molecule, and Pocket Grid C (with λ_geom_ = 3.0)^a^

Configuration	
lambda weights	[3.0, 1.0, 1.0, 1.0]
number of reads	500
number of sweeps	10000
Problem Size	
nonzero linear terms	770
nonzero quadratic terms	165 382
total nonzero terms	166 152
selected couplers	{(3, 1), (10, 13), (11, 8), (0, 3), (9, 9), (12, 6), (13, 7)}
	{(1, 2), (2, 4), (7, 10), (4, 5), (5, 0), (6, 11), (8, 12)}
Energies	
best energy	–4.626
geometric energy	–0.092
vdW energy	–3.057
electrostatic energy	1.086
residual energy	–2.664

a Execution time: 24.23 s.

To evaluate the specific role of the residual contribution, we repeated the calculation with that specific term switched off. When λ_residual_ = 0, the ligand is placed in a different region of the grid, despite the geometric term being enforced with λ_geom_ = 3 (Table 9). A further run with λ_geom_ = 1 and λ_residual_ = 0 (Table 10) shows that the ligand does not localize in a configuration consistent with favorable π-stacking. These comparisons indicate that the residual term is crucial for correctly steering the ligand toward the expected aromatic contact region.

**Table 9:** Results of the Annealing Computation Using a Benzene Ligand, a Tyrosine Molecule, and Pocket Grid C (with λ_geom_ = 3.0 and λ_mol_ = 0.0)^a^

Configuration	
lambda weights	[3.0, 1.0, 1.0, 0.0]
number of reads	500
number of sweeps	10000
Problem Size	
nonzero linear terms	770
nonzero quadratic terms	165 379
total nonzero terms	166 149
selected couplers	{(5, 41), (3, 46), (0, 44), (10, 50), (11, 49), (13, 48), (12, 47)}
	{(8, 51), (1, 45), (7, 53), (2, 43), (4, 42), (6, 52), (9, 54)}
Energies	
best energy	–3.128
geometric energy	0.094
vdW energy	–2.786
electrostatic energy	–0.351
residual energy	0.000

a Execution time: 24.87 s.

**Table 10:** Results of the Annealing Computation Using a Benzene Ligand, a Tyrosine Molecule, and Pocket Grid C (with λ_geom_ = 1.0 and λ_mol_ = 0.0)^a^

Configuration	
lambda weights	[1.0, 1.0, 1.0, 0.0]
number of reads	500
number of sweeps	10000
Problem Size	
nonzero linear terms	770
nonzero quadratic terms	165 379
total nonzero terms	166 149
selected couplers	{(9, 50), (0, 46), (10, 52), (11, 51), (2, 45), (12, 35), (13, 48)}
	{(1, 34), (3, 42), (4, 44), (5, 43), (6, 54), (7, 14), (8, 53)}
Energies	
best energy	–0.941
geometric energy	3.019
vdW energy	–2.646
electrostatic energy	–1.313
residual energy	0.000

a Execution time: 25.02 s

### Phenylalanine-Benzene

The same analysis was repeated using a phenylalanine fragment as pocket. The results with the residual term active and λ_geom_ = 3 are reported in Table 11. As in the tyrosine case, the ligand adopts a configuration that maximizes the residual contribution, which again plays a decisive role in determining the optimal pose.

**Table 11:** Results of the Annealing Computation Using a Benzene Ligand, a Phenylalanine Molecule, and Pocket Grid C (with λ_geom_ = 3.0)^a^

Configuration	
lambda weights	[3.0, 1.0, 1.0, 1.0]
number of reads	500
number of sweeps	10000
Problem Size	
nonzero linear terms	770
nonzero quadratic terms	165 381
total nonzero terms	166 151
selected couplers	{(11, 8), (0, 3), (1, 4), (12, 6), (13, 7), (10, 9), (2, 2)}
	{(3, 5), (7, 12), (4, 1), (5, 0), (9, 13), (6, 11), (8, 10)}
Energies	
best energy	–4.701
geometric energy	–0.092
vdW energy	–3.658
electrostatic energy	0.643
residual energy	–2.296

a Execution time: 24.86 s.

When the residual term is set to zero (Table 12), the minimum-energy configuration changes substantially, confirming that the π-stacking term is not redundant with respect to the other energetic contributions and is required to reproduce the expected binding geometry.

**Table 12:** Results of the Annealing Computation Using a Benzene Ligand, a Phenylalanine Molecule, and Pocket Grid C (with λ_geom_ = 3.0 and λ_mol_ = 0.0)^a^

Configuration	
lambda weights	[3.0, 1.0, 1.0, 0.0]
number of reads	500
number of sweeps	10000
Problem Size	
nonzero linear terms	770
nonzero quadratic terms	165 379
total nonzero terms	166 149
selected couplers	{(5, 41), (3, 46), (0, 44), (10, 50), (11, 49), (13, 48), (12, 47)}
	{(8, 51), (1, 45), (7, 53), (2, 43), (4, 42), (6, 52), (9, 54)}
Energies	
best energy	–2.890
geometric energy	0.009
vdW energy	–2.745
electrostatic energy	–0.155
residual energy	0.000

a Execution time: 24.94 s

#### Tryptophan-Benzene

Finally, we considered a tryptophan pocket. With λ_geom_ = 3.0 and a nonzero latent term, the optimal configuration reported in Table 13 again places the benzene ligand in a geometry compatible with strong π-stacking. In contrast, when the residual term is removed (Table 14), the ligand adopts a different pose and the total energy increases.

**Table 13:** Results of the Annealing Computation Using a Benzene Ligand, a Tryptophan Molecule, and Pocket Grid C (with λ_geom_ = 3.0)^a^

Configuration	
lambda weights	[3.0, 1.0, 1.0, 1.0]
number of reads	100
number of sweeps	2000
Problem Size	
nonzero linear terms	770
nonzero quadratic terms	165 382
total nonzero terms	166 152
selected couplers	{(0, 0), (2, 5), (11, 11), (10, 12), (3, 2), (7, 9), (12, 6), (13, 7)}
	{(1, 1), (8, 13), (4, 4), (5, 3), (6, 8), (9, 10)}
Energies	
best energy	–4.347
geometric energy	0.009
vdW energy	–3.276
electrostatic energy	1.316
residual energy	–2.391

a Execution Time: 25.03 s.

**Table 14:** Results of the Annealing Computation Using a Benzene Ligand, a Tryptophan Molecule, and Pocket Grid C (with λ_geom_ = 1.0 and λ_mol_ = 0.0)^a^

Configuration	
lambda weights	[3.0, 1.0, 1.0, 0.0]
number of reads	100
number of sweeps	2000
Problem Size	
nonzero linear terms	770
nonzero quadratic terms	165 379
total nonzero terms	166 149
selected couplers	{(5, 42), (6, 53), (3, 43), (0, 45), (10, 49), (2, 46), (11, 50), (13, 48)}
	{(12, 47), (9, 16), (1, 44), (4, 41), (8, 54), (7, 52)}
Energies	
best energy	–1.817
geometric energy	0.451
vdW energy	–2.581
electrostatic energy	0.313
residual energy	0.000

a Execution time: 25.32 s.

Taken together, these results demonstrate that the residual term plays a central role in determining the spatial arrangement of the ligand aromatic fragment, even in small and controlled systems. The combined evidence across benzene, tyrosine, phenylalanine, and tryptophan supports the relevance of explicitly modeling π-stacking interactions within the QUBO-based docking framework.

## Conclusions

In this work, we investigated the mathematical modeling of molecular interactions within a discrete Hamiltonian framework, explicitly written in QUBO form, in which all interaction terms and constraints are expressed using binary variable. This formulation is naturally compatible with quantum annealing algorithms, which are designed to solve combinatorial optimization tasks, and was here applied to the molecular docking problem, where the objective is to identify the lowest-energy conformation of a ligand in the binding pocket of a target protein.

The study focused specifically on noncovalent π-stacking interactions between aromatic rings. The central goal was to define a Hamiltonian that is both mathematically well-posed and physically meaningful, capable of encoding the essential geometric and energetic features of π-stacking in a form suitable for being encoded in graph-based molecular representations. To inform and calibrate the model, quantum-chemical calculations were performed on representative benzene dimer systems. These calculations provided reference energy landscapes used to tune the structure and coefficients of the Hamiltonian. The final formulation extends the geometric model proposed by Triuzzi et al.^11^ by introducing a new interaction term that captures the distance and orientation-dependence of π-stacking, encoded through a residual energy contribution extracted from ab initio data.

The resulting QUBO Hamiltonian was tested using classical simulated annealing on a series of controlled docking scenarios involving benzene ligands and aromatic residues (tyrosine, phenylalanine, and tryptophan). In all cases, the residual term derived from the latent energy was shown to play a decisive role in steering the ligand toward configurations consistent with stable π-stacking contacts, while the absence of this term led systematically to different, less physically meaningful minima. These results support the validity of the proposed parametrization and demonstrate that the additional term is not redundant with respect to standard electrostatic and van der Waals contributions.

A key challenge that emerged throughout the study concerns the trade-off between model accuracy and computational cost. Unlike the formulation by Triuzzi et al.,^11^ the present model explicitly includes hydrogen atoms in the optimization, increasing the chemical realism of the interaction description but also substantially enlarging the number of binary variables and quadratic couplings. This, in turn, makes the annealing procedure more computationally demanding and complicates the search for global minima in larger pocket grids. Future work will therefore explore simplified yet chemically consistent formulations that retain predictive power while reducing the overall resource requirements, for example through coarse-grained representations or effective potentials.

Several natural extensions of this work remain to be explored. First, a more systematic investigation of the energy dependence on rotational degrees of freedom—beyond the equilibrium-based corrections adopted here—would provide a more detailed and quantitative foundation for the angular components of the model. Second, while the present study deliberately focuses on a classical optimization backend to validate the proposed Hamiltonian in a controlled setting, the formulation has been explicitly designed to be compatible with quantum annealing architectures. Its deployment on quantum annealing hardware, together with applications to larger and more complex ligand–receptor systems, therefore represents a natural extension aimed at exploring scalability and hardware-specific effects, rather than a prerequisite for the validity of the current results.

Overall, the results indicate that discrete Hamiltonians in QUBO form, when grounded in high-quality physical data and carefully structured, offer a promising mathematical framework for approximating molecular interactions in docking problems. From a broader perspective, this work illustrates how graph-based modeling, empirical parametrization, and discrete optimization can be combined to address relevant questions in molecular recognition and computational chemistry, and provides a foundation for further developments in quantum-inspired and quantum-enabled approaches to molecular modeling.

## Data Availability

The code used in this study is publicly available at GitHub: https://github.com/alebeneventi/Pi-Stacking/tree/main
